# The Mitogen‐Activated Protein Kinase GhNTF3 Modulates the Arbuscular Mycorrhizal Symbiosis‐Immunity Trade‐Off in Cotton by Regulating Salicylic Acid Biosynthesis

**DOI:** 10.1002/advs.77315

**Published:** 2026-08-25

**Authors:** Shuangjie Jia, Jingshang Wen, Jihang Li, Meina Feng, Yiming He, Ruojun Liu, Qian Liu, Junli Zhang, Kai Cheng, Xiangyu Zhang, Xiao Zhang

**Affiliations:** ^1^ State Key Laboratory of Cotton Bio‐Breeding and Integrated Utilization School of Life Sciences Henan University Kaifeng China; ^2^ College of Life Sciences Henan Agricultural University Zhengzhou Henan China; ^3^ Institute of Edible Fungi Henan Academy of Agricultural Sciences Zhengzhou China

**Keywords:** arbuscular mycorrhiza, mitogen‐activated protein kinase, phosphoproteome, salicylic acid, Verticillium wilt

## Abstract

Plant immunity is essential for survival against pathogen invasion. However, enhanced immune activation can restrict arbuscular mycorrhizal (AM) fungal colonization. Therefore, plants must finely balance immunity and symbiosis to optimize fitness, but the key regulators underlying this trade‐off remain unclear. Here, we identify a novel mitogen‐activated protein kinase, GhNTF3, as a central regulator of the AM symbiosis‐immunity balance. Knockdown of *GhNTF3* promotes AM symbiosis but decreases Verticillium wilt resistance in cotton. *GhNTF3* overexpression suppresses AM symbiosis. GhNTF3 interacts with GhWAK13, a previously characterized wall‐associated kinase that is specifically induced by AM symbiosis, at the plasma membrane. Genetic evidence indicates that *GhWAK13* functions in association with *GhNTF3* during AM symbiosis. *GhWAK13* negatively regulates salicylic acid (SA) accumulation, whereas GhNTF3 positively regulates SA accumulation during AM symbiosis. Knockdown of SA biosynthesis genes or the SA receptor gene *GhNPR1* significantly enhanced AM fungal colonization, whereas exogenous SA application strongly inhibited symbiosis. Moreover, GhNTF3 interacts with GhJAZ6 in the nucleus to enhance Verticillium wilt resistance, potentially through SA accumulation in cotton. Collectively, our findings identify a molecular module in which the interplay between GhNTF3 and GhWAK13 dynamically balances AM symbiosis and Verticillium wilt resistance through antagonistic regulation of the SA signaling pathway.

## Graphical abstract

AM symbiosis induces GhWAK13 to promote fungal colonization. *GhNTF3* negatively regulates symbiosis by promoting SA biosynthesis. GhWAK13 sequesters GhNTF3 at the membrane, limiting its nuclear translocation and relieving SA‐mediated suppression. Under Verticillium stress, GhNTF3 enters the nucleus and interacts with GhJAZ6 to activate SA signaling, functioning as a molecular switch balancing symbiosis and immunity.

## 1 Introduction

Arbuscular mycorrhizal (AM) fungi form mutually beneficial symbiotic relationships with over 80% of terrestrial plant species, including industrial crops such as cotton (*Gossypium* spp.) [1, 2]. Under phosphate‐deficient conditions, plant roots exude strigolactones (SLs) into the rhizosphere [3]. The germination of AM fungal spores is induced by SLs, which subsequently promote hyphal branching. The AM fungal hyphae penetrate the root cell wall and extend into the root apoplastic space, forming highly branched arbuscular structures. In return, the root plasma membrane develops into a periarbuscular membrane (PAM) that envelops the arbuscule, collectively establishing the symbiotic interface between AM fungi and the host plant [1, 4]. AM fungi facilitate the uptake of mineral nutrients, especially phosphate and nitrogen, in exchange for plant‐derived carbohydrates [5]. Beyond improving nutrient acquisition, AM symbiosis contributes to enhanced tolerance to abiotic stresses such as drought and salinity and can confer increased resistance to certain pathogens [6, 7]. As a result, AM symbiosis has attracted significant interest as a potential strategy for improving crop productivity in sustainable agriculture.

Plants establish mutualistic symbiosis with AM fungi to acquire essential nutrients, while simultaneously activating immune defense systems to fend off soil‐borne pathogens throughout their life cycle [8, 9]. Plants have evolved two major layers of immunity to resist pathogen attack or shape microbe colonization. The first layer is mediated by pattern recognition receptors (PRRs) that are located in the plasma membrane to perceive pathogen‐associated molecular patterns (PAMPs) or microbe‐associated molecular patterns (MAMPs) to activate pattern‐triggered immunity (PTI) [10]. For example, the pathogen of cotton Verticillium wilt, *Verticillium dahliae*, releases chitin onto the plant root surface, which is recognized by chitin elicitor receptor kinase 1 (CERK1) to trigger immunity responses [11]. Once the pathogen successfully infects plants, plants can activate the second layer of immunity named effector‐triggered immunity (ETI) through recognizing effector produced by pathogens [12].

Mitogen‐activated protein kinase (MAPK) cascades are central regulators of plant immunity, orchestrating defense responses upon pathogen perception. Upon the activation of PTI/ETI, MAPKs such as MPK3 and MPK6 are rapidly phosphorylated [13, 14, 15], initiating early immune responses. Subsequently, the MAPK cascade activates late‐phase immune responses, including the transcriptional regulation of defense‐related genes [16, 17] and biosynthesis of defense hormones [18]. Salicylic acid (SA) serves as a pivotal signaling molecule in plant immunity [19]. SA plays a central role in systemic acquired resistance (SAR), primarily limiting infections by biotrophic/hemibiotrophic pathogens and modulating symbiotic interactions [20]. The SA biosynthesis pathway is governed by two key rate‐limiting enzymes: isochorismate synthase 1 (ICS1) and phenylalanine ammonia‐lyase 1 (PAL1) [12, 21]. Additionally, NONEXPRESSOR OF PATHOGENESIS‐RELATED GENES 1 (NPR1) functions as both a central regulator and SA receptor in the SA signaling pathway. However, the potential involvement of MAPK signaling and downstream SA pathways in regulating AM symbiosis remains unclear.

Moreover, excessive activation of defense responses can hinder AM symbiosis [22, 23]. The establishment of AM symbiosis requires plants to partially suppress their immune responses to allow AM fungal entry and colonization [24]. This process involves symbiosis‐defense trade‐offs [25]. In cotton, a globally important fiber and oilseed crop, AM symbiosis has likely been preserved throughout domestication due to its agronomic benefits [2]. Cotton also needs to maintain defense responses during production to resist damage caused by pathogens such as *Verticillium dahliae* [26]. Understanding the molecular mechanisms that allow cotton to regulate this symbiosis‐defense trade‐off is thus of both fundamental and agronomic significance.

Previously, we found that GhWAK13, a cotton wall‐associated kinase, promotes AM colonization and weakens immune responses [2]. However, the upstream or antagonistic signaling components that modulate GhWAK13 function remained unknown. Here, we report the identification of a novel MAP kinase, GhNTF3, which suppresses AM colonization while enhancing SA‐dependent immunity. We demonstrate that GhNTF3 physically interacts with GhWAK13 and functions antagonistically to GhWAK13. *GhWAK13* restrains *GhNTF3*‐mediated immune activation to maintain a permissive state for AM symbiosis, whereas GhNTF3 feedback regulates *GhWAK13*‐mediated AM symbiosis to prevent excessive fungal colonization. Together, our findings uncover a GhNTF3‐GhWAK13 module that fine‐tunes the dynamic balance between symbiosis and immunity in cotton. This regulatory node enables the plant to prioritize mutualistic colonization under nutrient‐deficient conditions while retaining the capacity to activate immune responses under pathogen threat.

## 2 Results

### 2.1 Phosphoproteome Reveals the Down‐Regulation of Multiple Proteins Involved in MAPK Pathway in Response to AM Fungal Inoculation

**FIGURE 1:**
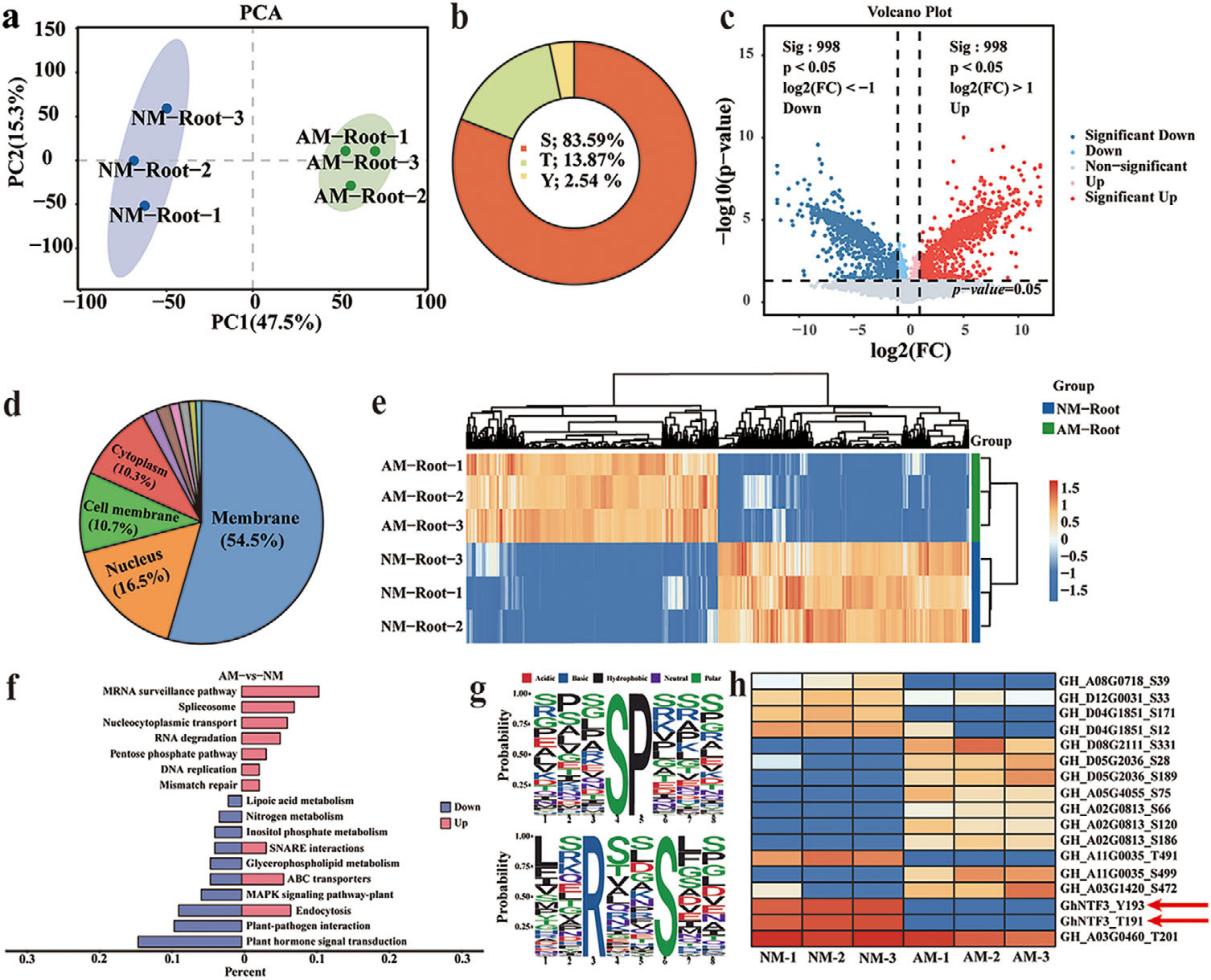
Effects of arbuscular mycorrhizal (AM) fungal inoculation on protein phosphorylation in cotton roots. (a) The principal component analysis (PCA) of mycorrhizal cotton samples. PC1 and PC2 indicate the first and second principal components derived from principal component analysis, respectively. *n* = 3. AM represents AM fungal inoculation, whereas NM represents the non‐inoculated control. PCA was performed based on the abundance profiles of differentially phosphorylated peptides. (b) Percentage distribution of protein phosphorylation sites in mycorrhizal cotton. S represents serine. T represents threonine. Y represents tyrosine. (c) Volcano plot analysis of differentially phosphoproteins in mycorrhizal cotton. Red dots represent up‐regulated phosphoproteins in AM fungal‐inoculated samples, while blue dots indicate down‐regulated phosphoproteins following AM fungal inoculation. *p* < 0.05. (d) Subcellular localization analysis of differentially phosphoproteins. (e) Heatmap analysis of differentially phosphorylated proteins. The normalized data are presented in red‐blue heatmap, where blue and red indicate down‐regulated and up‐regulated phosphoprotein under AM inoculation, respectively. *n* = 3. (f) Comparative KEGG enrichment analysis of up‐ and down‐regulated pathways. The bar plot displays the top 10 most significantly enriched KEGG pathways (ranked by *p*‐value) in both up‐regulated (red) and down‐regulated (blue) protein clusters. (g) Motif analysis of differential phosphorylation sites in mycorrhizal cottons. The *y*‐axis represents the relative frequency of amino acid occurrence. (h) Heatmap analysis of differentially phosphorylated MAPK proteins. Data were normalized using the mean value of each protein across all samples before heatmap visualization. The red arrow indicates the position of GhNTF3. *n* = 3.

Protein phosphorylation is a central process in plant‐microbe symbiosis. To study how AM fungal inoculation affects the cotton defense signaling pathway, DIA‐based phosphoproteomic analysis was performed using mycorrhizal cotton roots with 5 weeks post inoculation (Figure S1a,b). In general, 9397 different phosphopeptides were identified from mycorrhizal roots. Root sample clustering analysis revealed that AM fungus‐colonized roots and non‐inoculated controls were separated along principal component 1 (47.5%) (Figure 1a), indicating significant differences in protein phosphorylation. By analyzing phosphorylation site preferences in mycorrhizal roots, serine (83.59%) and threonine (13.87%) phosphorylation events were predominantly induced by AM symbiosis (Figure 1b). AM fungal inoculation significantly upregulated 998 phosphopeptides and downregulated 998 phosphopeptides (Figure 1c,e). The detailed phosphopeptides are listed in Dataset S1. Among these differentially regulated phosphopeptides that were induced by AM fungal inoculation, the corresponding proteins were primarily localized to membranes (54.5%), nucleus (16.5%), and cytoplasm (10.3%) (Figure 1d). KEGG enrichment analysis revealed significant downregulation of the plant hormone signaling, plant‐pathogen interaction, and MAPK signaling pathway following AM fungal inoculation (Figure 1 and Figure S2b), suggesting a negative regulation of these pathway during AM symbiosis. Additionally, AM fungal inoculation positively regulated several pathways, including the mRNA surveillance pathway and spliceosome (Figure 1f and Figure S2a). Motif analysis revealed that serine‐proline (SP) and arginine‐x‐x‐serine (R‐x‐x‐S) motifs were predominantly induced by AM fungal inoculation (Figure 1g). Consequently, we identified differentially phosphorylated MAPKs from phosphopeptides, revealing that two phosphorylation sites (T191 and Y193) of GhNTF3 (a MAP kinase) were significantly downregulated by AM fungal inoculation (Figure 1h). Through pathway and protein‐protein interaction (PPI) analysis (Figures S3 and S4), GhNTF3 may play a central role in the regulation of AM symbiosis.

### 2.2 *GhNTF3* Negatively Regulated AM Symbiosis in Cotton

**FIGURE 2:**
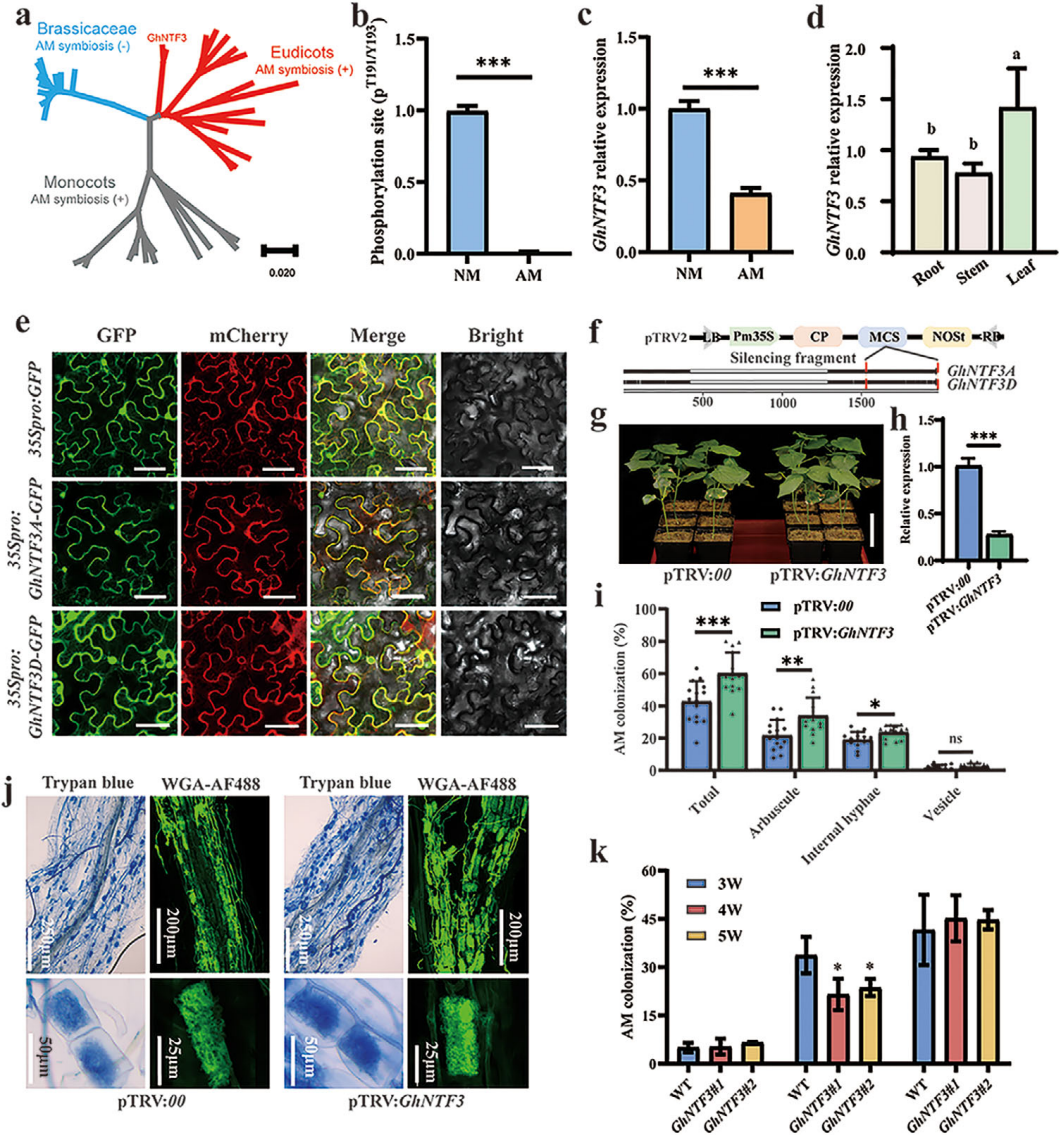
Functional characterization of GhNTF3 in mycorrhizal cottons. (a) Phylogenetic analysis of GhNTF3 protein from plant species with (+) or (−) AM symbiosis. AM symbiosis (−) represents plants incapable of forming symbiotic associations with AM fungi, whereas AM symbiosis (+) refers to those capable of establishing AM symbiosis. Scale bar represents 0.1 amino acid substitutions per site. (b) Effect of AM fungi inoculation on GhNTF3 phosphorylation. Error bars indicate ±SD of independent replicates. Two‐tailed Student's *t*‐test was used to assess statistical differences between AM and NM groups. *n* = 3. ^***^, *p* < 0.001. (c, d) Expression patterns of *GhNTF3* in response to AM symbiosis (c), and in different tissues (d). Error bars indicate ±SD of independent replicates. Two‐tailed Student's *t*‐test was used to assess statistical differences between AM and NM groups. ^***^, *p* < 0.001. *n* ≥ 4. *GhUBQ7* was used as a housekeeping gene in cotton. One‐way analysis of variance (ANOVA) was used to determine statistically significant differences among different tissues, followed by Tukey‐test post hoc analysis. Different letters indicate significant differences among different tissues. *p* < 0.05. (e) Subcellular localization of GhNTF3 protein. Bar = 50 µm. The mCherry channel shows the membrane localization observed in tobacco leaves after staining with the membrane dye FM4‐64. (f) Silencing fragment of *GhNTF3* used for VIGS. (g) Growth phenotype of *GhNTF3‐*silenced cottons under AM symbiosis. At least 15 independent cottons were used in this experiment. Each experiment was repeated three times. (h) Silencing efficiency of *GhNTF3* in mycorrhizal cottons. Error bars indicate ±SD of independent replicates. Two‐tailed Student's *t*‐test was used to assess statistical differences between pTRV:*00* and pTRV:*GhNTF3* plants. *n* = 4. ^***^, *p* < 0.001. (i, j) Effects of *GhNTF3* silencing on AM fungal colonization (i) and colonization structures of AM fungi (j) in cotton roots. Error bars indicate ±SD of independent replicates. Two‐tailed Student's *t*‐test was used to assess statistical differences between pTRV:*00* and pTRV:*GhNTF3* plants. The data points in the bar graphs in Figure 2i represent individual biological replicates. ^*^, *p* < 0.05; ^**^, *p* < 0.01; ^***^, *p* < 0.001, ns, not significant. *n* = 15. (k) Effect of *GhNTF3* overexpression on AM fungal colonization. Error bars indicate ±SD of independent replicates. Two‐tailed Student's *t*‐test was used to assess statistical differences between two groups. 3 W, 4 W, and 5 W represent samples collected post 3, 4, and 5 weeks after AM inoculation, respectively. *n* ≥ 3. ^*^, *p* < 0.05.

Phylogenetic analysis of GhNTF3 homologs across angiosperms revealed a distinct divergence into two clades: one comprising non‐AM Brassicaceae species and one AM‐colonizable clade (Figure 2a), suggesting lineage‐specific functional diversification of GhNTF3 related to symbiotic adaptation. The cotton genome contains two highly homologous (98.4%) GhNTF3 sequences (GhNTF3A and GhNTF3D), located on the At and Dt subgenomes, respectively. We considered *GhNTF3A* and *GhNTF3D* to represent the same gene, and *GhNTF3A* was used as the representative for subsequent analyses (Figure S5). GhNTF3 contains a S_TKc domain that spans amino acid residues 32 to 319, with a threonine‐glutamate‐tyrosine (TEY) motif located at positions 191 to 193 within the T‐loop activation region (Figure S6a,b). Both predicted phosphorylation sites and mass spectrometry analysis revealed that GhNTF3 is phosphorylated at T191 and Y193, and AM inoculation significantly reduces phosphorylation at these sites (Figure 2b and Figure S6b). The relative expression of *GhNTF3* was downregulated by AM inoculation (Figure 2c). Moreover, *GhNTF3* is highly expressed in cotton roots, stems, and leaves, with the highest expression in leaves (Figure 2d). To determine the subcellular localization of GhNTF3, GhNTF3A/D was fused with GFP at its C‐terminus and transient expressed in tobacco leaves. The GhNTF3A/D‐GFP fusion protein localized to both the nucleus and cytoplasm (Figure 2e), suggesting its functional significance associated with its subcellular localization.

To investigate the role of *GhNTF3* in AM symbiosis, homologous fragments of *GhNTF3* from both the At and Dt subgenomes were used to silence *GhNTF3A/D* (Figure 2f and Figure S5). Silencing of *GhNTF3* significantly increased plant height and biomass in mycorrhizal cotton (Figure 2g and Figure S7a,b,e), but had no significantly effects on leaf width‐to‐length or root‐to‐shoot ratio (Figure S7c,d). *GhNTF3* silencing increased leaf area and plant biomass in nonmycorrhizal cotton (Figure S8a–e). Silencing of *GhNTF3* significantly increased total AM colonization, arbuscule colonization, and internal hyphae colonization (Figure 2h,i). However, both trypan blue and WGA‐AF488 staining revealed that arbuscule development was not affected by *GhNTF3* silencing, although *GhNTF3* silencing dramatically enhanced arbuscule colonization density in cotton roots (Figure 2j). These results were consistent with the expression of AM symbiosis marker genes *GhPT5*, *GhBCP1*, and *GhABCG1*, as well as the housekeeping gene of AM fungi *RiEF1α* (Figure S9). The expression of pathogenesis‐related (PRs) genes was downregulated by *GhNTF3* silencing (Figure S10), suggesting that *GhNTF3* may positively regulate the immune response but negatively regulate AM symbiosis.

### 2.3 *GhNTF3* Positively Regulated Verticillium Wilt Resistance in Cotton

**FIGURE 3:**
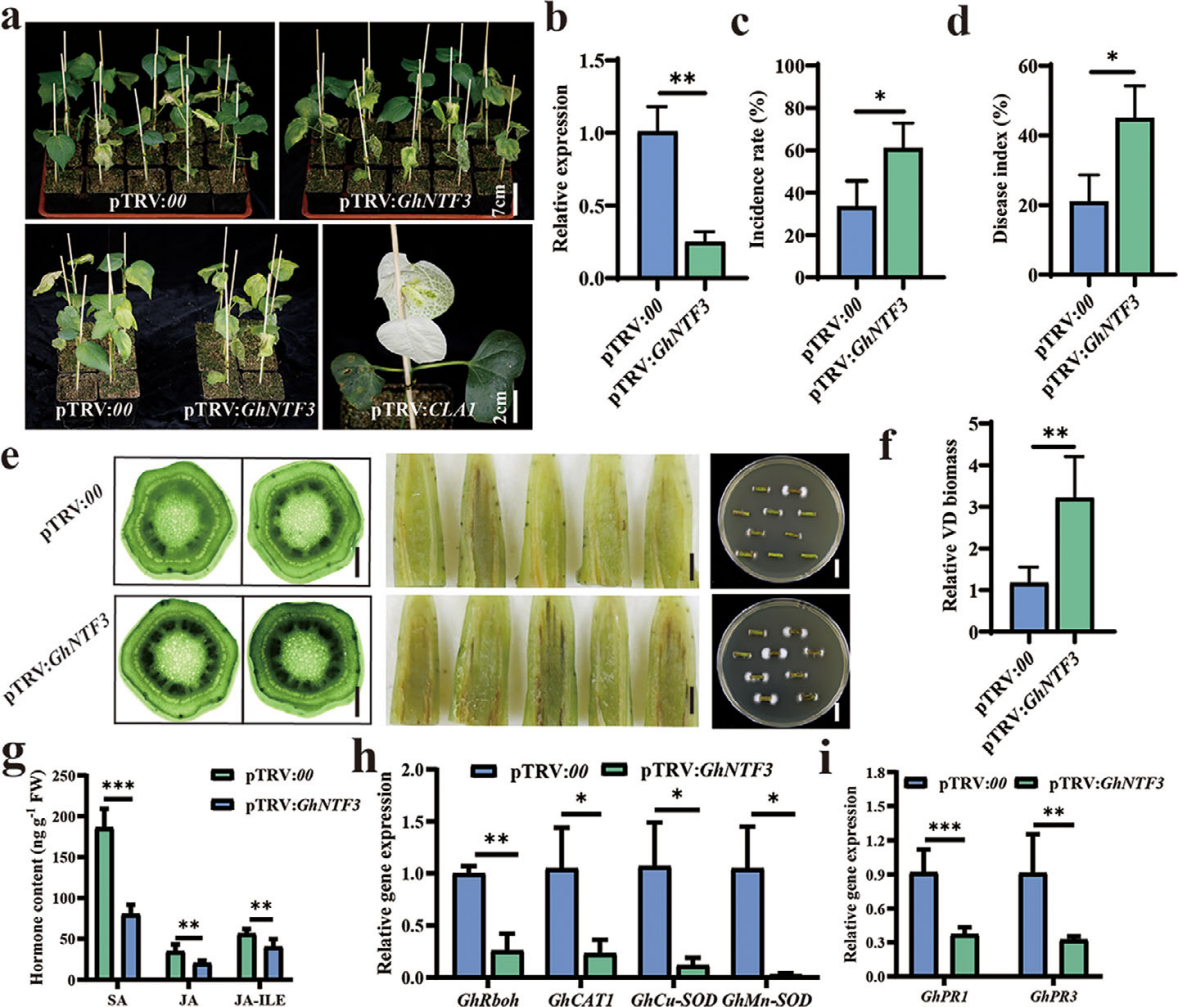
The role of *GhNTF3* in Verticillium wilt resistance in cottons. (a) Phenotypes of cotton seedlings 2 weeks post *V. dahliae* infection. At least 15 cotton seedlings were evaluated for Verticillium wilt resistance. Each experiment was repeated three times. pTRV:*GhCLA1* was used as a positive control. Scale bar = 2 cm. (b) Silencing efficiency of *GhNTF3* in cottons. Error bars indicate ±SD of independent replicates. Two‐tailed Student's *t*‐test was used to assess statistical differences between pTRV:*00* and pTRV:*GhNTF3* plants. *n* = 5. ^**^, *p* < 0.01. (c–i) Effects of *GhNTF3* silencing on incidence rate (c), disease index (d), degree of vascular browning and stem recovery culture (e), pathogen carrying capacity in stem (f), hormone content (g), and the expression of disease resistance‐related genes (h‐i). Error bars indicate ±SD of independent replicates. Two‐tailed Student's *t*‐test was used to assess statistical differences between pTRV:*00* and pTRV:*GhNTF3* plants. Sample sizes were *n* = 3 for disease index and incidence rate, and *n* ≥ 3 for hormone and gene expression analyses. ^*^, *p* < 0.05; ^**^, *p* < 0.01; ^***^, *p* < 0.001. Scale bars represent 500 µm (vascular browning), 2.5 mm (stem sections), and 1 cm (stem recovery culture).

Previous results indicated that *GhNTF3* regulates the expression of defense genes, suggesting that *GhNTF3* may be involved in the regulation of cotton immunity (Figure S10). Verticillium wilt is the “cancer” of cotton production. After infection with *V. dahliae*, leaves of *GhNTF3*‐silenced cotton exhibited more severe yellowing, wilting, and even death compared with the control (Figure 3a). Silencing of *GhNTF3* significantly increased the disease index and incidence rate of Verticillium wilt in cotton in three independent experiments (Figure 3b–d). Meanwhile, we observed more severe browning in vascular tissue of *GhNTF3*‐silenced cotton compared with the control (Figure 3e). Both the *V. dahliae* fungal biomass assay and stem recovery culture experiments confirmed that *GhNTF3* silencing increased the spread of *V. dahliae* from roots to stems (Figure 3e,f). SA and JA are major hormones involved in plant defense responses. Silencing of *GhNTF3* decreased SA, JA, and JA‐ILE levels by 56.8%, 40.8%, and 28.3%, respectively (Figure 3g). *GhNTF3* positively regulated the expression of defense genes such as *GhRbohD*, *GhCAT1*, *GhCu‐SOD*, *GhMn‐SOD* in cotton roots. The expression of *PRs* genes such as *GhPR1* and *GhPR3* was downregulated by *GhNTF3* knockdown (Figure 3h,i). Upon treatment with pathogen‐derived chitin, silencing of *GhNTF3* markedly suppressed the transcription of reactive oxygen species (ROS)‐scavenging genes, including *GhCAT1* and *GhCu‐SOD1* (Figure S11). Together, these findings establish GhNTF3 as a positive regulator of Verticillium wilt resistance in non‐AM cotton.

### 2.4 GhNTF3 Interacts With the AM Symbiosis‐Positive Regulator GhWAK13

**FIGURE 4:**
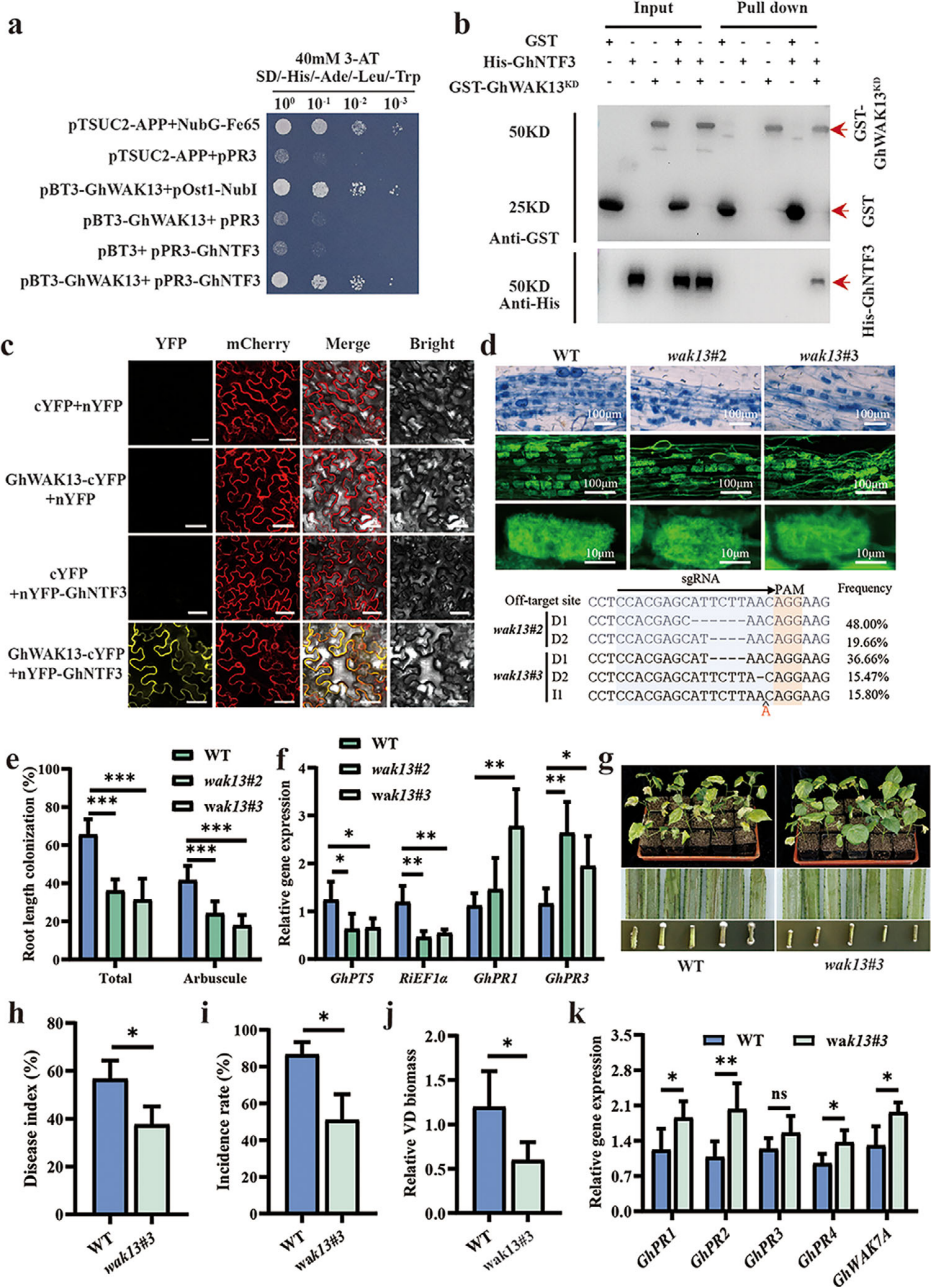
Interaction between GhNTF3 and GhWAK13. (a) GhNTF3 interacts with GhWAK13 in the yeast two‐hybrid system. 40 mm 3‐AT was used to suppress false‐positive results. (b) GhNTF3 interacts with the kinase domain of GhWAK13 in vitro. GST protein was used as a negative control. (c) GhNTF3 interacts with GhWAK13 on the plasma membrane. Bar = 50 µm. The mCherry channel shows the membrane localization observed in tobacco leaves after staining with the membrane dye FM4‐64. (d) AM fungal colonization in roots of *GhWAK13*‐knockout lines. Scale bars are shown directly in the images. *wak13#2* and *wak13#3* were used to observe AM fungal colonization under both trypan blue and WGA‐AF488 stains. Genome editing profiles of these two lines are displayed below the phenotypes of AM fungal colonization. (e) Root length colonization in *GhWAK13*‐knockout cotton. Error bars indicate ±SD of independent replicates. Two‐tailed Student's *t*‐test was used to assess statistical differences between WT and *wak13* plants. *n* = 9. ^**^, *p* < 0.01; ^***^, *p* < 0.001. (f) Expression of AM symbiotic marker genes in *GhWAK13*‐knockout cottons. *GhUBQ7* was used as a housekeeping gene in cotton. Error bars indicate ±SD of independent replicates. Two‐tailed Student's *t*‐test was used to assess statistical differences between WT and *wak13* plants. *n* = 5. (g) Effect of *GhWAK13*‐knockout on Verticillium wilt resistance in cotton. At least 15 cotton seedlings were investigated for Verticillium wilt resistance. Each experiment was repeated three times. (h–k) Effects of *GhWAK13*‐knockout on the disease index (h), incidence rate (i), relative VD biomass (j), and defense gene expression (k) of Verticillium wilt. Error bars indicate ±SD of independent replicates. Two‐tailed Student's *t*‐test was used to assess statistical differences between WT and *wak13* plants. For relative gene expression, *n* = 5. ^*^, *p* < 0.05; ^**^, *p* < 0.01; ns, not significant.

Given the critical role of *GhNTF3* in AM symbiosis, we performed a yeast two‐hybrid screen of an AM root libraries using GhNTF3 as bait and identified GhWAK13, a wall‐associated kinase, as an interacting partner (Table S1). Further verification using a yeast two‐hybrid membrane system confirmed that GhNTF3 interacts with the full‐length GhWAK13 (Figure 4a). The kinase domain of GhWAK13 and GhNTF3 directly interact in vitro using a GST pull‐down and Y2H assays (Figure 4b and Figure S12). We observed that GhNTF3 and GhWAK13 interact at the plasma membrane in tobacco leaves using bimolecular fluorescence complementation (Figure 4c). However, we failed to obtain positive results from the co‐immunoprecipitation (Co‐IP) experiments between GhNTF3 and GhWAK13 (Figure S13), primarily because overexpression of *GhWAK13* caused tobacco leaf necrosis, making it impossible to detect sufficient GhWAK13 protein levels.

The *GhWAK13*‐knockout cotton was obtained using CRISPR‐Cas9 technology, with editing efficiencies of 67.66% for *wak13*#2 and 67.93% for *wak13*#3 (Figure 4d). Knocking out of *GhWAK13* significantly reduced root length colonization by AM fungi and decreased arbuscule density in cotton roots (Figure 4d,e). This result was consistent with the expression of the AM fungal marker gene *GhPT5* and the AM fungal housekeeping gene *RiEF1α*. Additionally, the *PR* genes *GhPR1* and *GhPR3* were upregulated in *GhWAK13*‐knockout plants (Figure 4f), suggesting that *GhWAK13* may regulate AM symbiosis through SA signaling. *GhWAK13*‐knockout plants showed enhanced resistance to Verticillium wilt compared with the wildtype (Figure 4g). The disease index, incidence rate, and relative VD biomass were reduced in *GhWAK13*‐knockout plants (Figure 4h–j). The expression of *GhPR2*, *GhPR3, GhPR4*, and *GhWAK7A* was negatively regulated by *GhWAK13* during AM symbiosis (Figure 4k).

### 2.5 Transcriptome and Metabolome Analyses Reveal Antagonistic Regulation of SA Accumulation by *GhNTF3* and *GhWAK13* in Cotton Roots

Since *GhNTF3* acts as a negative regulator of AM symbiosis but a positive regulator of plant immunity, we further performed transcriptomic and metabolomic profiling of *GhNTF3*‐silenced cotton roots to investigate its regulatory roles in these processes. Transcriptomic analysis revealed that *GhNTF3* silencing significantly altered gene expression of AM roots, with 479 genes upregulated and 289 downregulated (Figure S14a). Integrative analysis with previously identified AM symbiosis‐ and *V. dahliae*‐responsive genes showed that 273 *GhNTF3*‐downregulated genes overlapped with AM‐induced genes, whereas 116 *GhNTF3*‐upregulated genes overlapped with *V. dahliae*‐induced genes (Figure S14b,c). However, only 16 *GhNTF3*‐downregulated genes were not associated with AM‐induced genes, indicating a strong association between GhNTF3 and AM symbiosis (Figure S14b). KEGG pathway analysis revealed that the 273 downregulated genes were mainly enriched in fatty acid metabolism, fatty acid biosynthesis, and biotin metabolism (Figure S14d), while the 116 upregulated genes overlapping with the *V. dahliae* response were enriched in phenylpropanoid biosynthesis, taurine and hypotaurine metabolism, and secondary metabolite biosynthesis (Figure S14e). To validate the transcriptomic data, we examined hormone‐related genes by qRT‐PCR. *GhNTF3* silencing markedly suppressed salicylic acid biosynthesis genes (*GhPAL10* and *GhICS1*) but induced jasmonic acid biosynthesis genes (*GhLOX1.5* and *GhOPR3*) (Figure S14f), consistent with the transcriptomic results.

**FIGURE 5:**
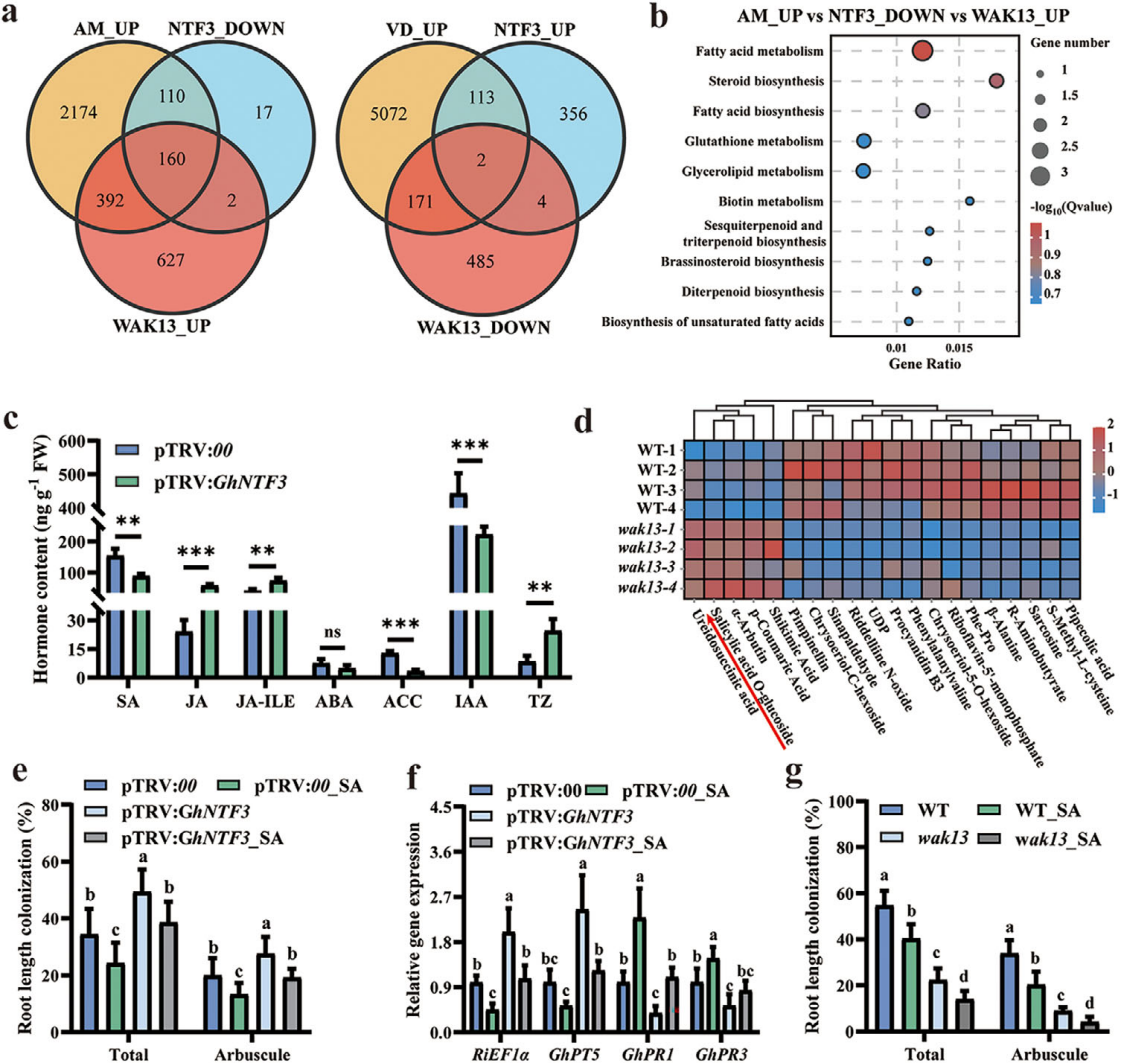
Transcriptomic and metabolomic analyses reveal the involvement of *GhWAK13* and *GhNTF3* in SA biosynthesis during AM symbiosis. (a) Venn analysis of cotton root transcriptomes under AM fungus symbiosis, *V. dahliae* infection, *GhNTF3* silencing, and *GhWAK13* knockout. AM_UP represents genes upregulated during AM symbiosis. GhNTF3_DOWN represents genes upregulated by *GhNTF3* silencing. GhNTF3_UP represents genes downregulated by *GhNTF3* silencing. VD_UP represents genes upregulated by *V*. *dahliae* infection. WAK13_UP represents genes upregulated by *GhWAK13* knockout. WAK13_DOWN represents genes downregulated by *GhWAK13*‐knockout. (b) KEGG enrichment analysis of co‐regulated genes under AM fungal symbiosis, *GhNTF3* silencing, and *GhWAK13* knockout. The Top 10 significantly enriched KEGG pathways are shown. (c) Effect of *GhNTF3* silencing on hormone contents in AM roots. SA, salicylic acid; JA, jasmonic acid; JA‐Ile, jasmonoyl‐isoleucine; ABA, abscisic acid; ACC, 1‐aminocyclopropane‐1‐carboxylic acid; IAA, indole‐3‐acetic acid; TZ, zeatin. Two‐tailed Student's *t*‐test was used to assess statistical differences between pTRV:*00* and pTRV:*GhNTF3* plants. Error bars indicate ±SD of independent replicates. *n* ≥ 3. ^**^, *p* < 0.01; ^***^, *p* < 0.001. ns, not significant. (d) Metabolic analysis of *GhWAK13* knockout roots. Data were normalized using the mean value of each metabolite across all samples before heatmap visualization. *n* = 4. The red arrow represents the position of salicylic acid O‐glucoside. (e, f) Effects of exogenous SA application on AM symbiosis (e) and relative gene expression (f) in *GhNTF3*‐silenced cottons. pTRV:*00*_SA and pTRV:*GhNTF3*_SA indicate pTRV:*00* and pTRV:*GhNTF3* plants, respectively, subjected to exogenous SA treatment. One‐way ANOVA was used to determine statistically significant differences among treatments, followed by Tukey‐test post hoc analysis. Each parameter was analyzed separately using ANOVA. Different letters indicate significant differences among treatments. *p* < 0.05. Sample sizes were *n* = 9 for root length colonization, and *n* = 5 for gene expression analyses. (g) Effect of exogenous SA application on AM symbiosis in *GhWAK13* mutant cottons. WT_SA and *wak13*_SA indicate WT and *wak13* plants, respectively, subjected to exogenous SA treatment. One‐way ANOVA was used to determine statistically significant differences among treatments, followed by Duncan‐test post hoc analysis. Each parameter was analyzed separately using ANOVA. Different letters indicate significant differences among treatments. *n* = 9. *p* < 0.05.

Integrated transcriptomic analysis of *GhWAK13* and *GhNTF3* revealed two distinct gene regulatory patterns. A total of 160 genes were positively regulated by both *GhWAK13* and AM fungal inoculation but negatively regulated by *GhNTF3* (Figure 5a). KEGG analysis revealed that these 160 genes were enriched in biological processes such as fatty acid metabolism, steroid biosynthesis, and fatty acid biosynthesis (Figure 5b). However, the intersection between *GhWAK13*‐downregulated and *GhNTF3*‐upregulated genes contained only 6 genes, suggesting that *GhWAK13* and *GhNTF3* function through largely independent pathway in regulating Verticillium wilt resistance (Figure 5a). Hormone profiling showed that *GhNTF3* positively regulated SA content but negatively regulated JA and JA‐Ile contents in AM roots (Figure 5c). In non‐mycorrhizal roots, silencing of *GhNTF3* led to a decrease in SA levels and an increase in JA‐Ile content, whereas JA levels were not significantly affected by *GhNTF3* silencing (Figure S15). Meanwhile, metabolomic analysis showed that *GhWAK13* negatively regulates SA‐O‐glucoside, coumaric acid, shikimic acid, and ureidosuccinic acid, while positively modulating S‐methyl‐L‐cysteine, pipecolic acid, and sarcosine (Figure 5d). Quantitative analysis of SA content in *ghwak13* mutant indicated that *GhWAK13* negatively regulates SA accumulation in AM roots (Figure S16). Exogenous SA application suppressed the enhanced AM symbiosis observed in *GhNTF3*‐silenced plants (Figure 5e), consistent with the expression patterns of the AM symbiosis marker *GhPT5* and the SA signaling markers *GhPR1* and *GhPR3* (Figure 5f). In contrast, exogenous SA treatment further reduced AM fungal colonization in *ghwak13* mutant (Figure 5g and Figure S17). These results suggest that SA partially mediates the role of *GhWAK13* in regulating AM symbiosis.

### 2.6 Altered Salicylic Acid Levels Affect AM Fungal Colonization in Cotton

**FIGURE 6:**
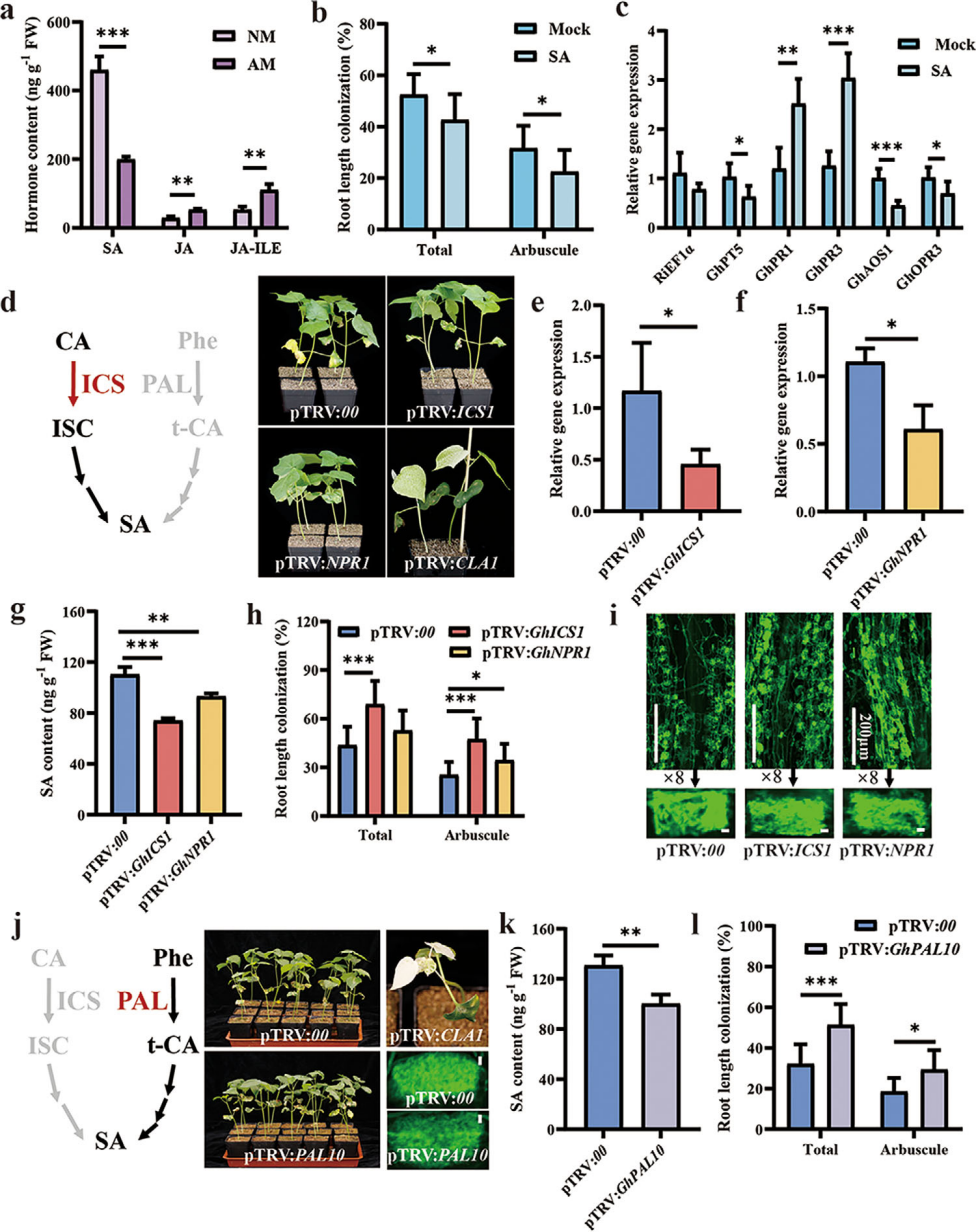
Effects of salicylic acid on AM symbiosis in cotton. (a) Effects of AM fungal inoculation on salicylic acid and jasmonic acid contents in cotton roots. SA, salicylic acid; JA, jasmonic acid; JA‐Ile, jasmonoyl‐isoleucine. Two‐tailed Student's *t*‐test was used to assess statistical differences between NM and AM plants. Error bars indicate ±SD of independent replicates. *n* = 3. ^**^, *p* < 0.01; ^***^, *p* < 0.001. (b, c) Effects of exogenous SA application on AM fungal colonization (b) and gene expression (c). SA represents exogenous SA application. Two‐tailed Student's *t*‐test was used to assess statistical differences between mock‐ and SA‐treated plants. Error bars indicate ±SD of independent replicates. Sample sizes were *n* = 9 for root length colonization, and *n* = 5 for gene expression analyses. *GhUBQ7* was used as a housekeeping gene in cotton. 2^(‐△△ct)^ methods was used to calculate the relative expression levels of these genes. ^*^, *p* < 0.05; ^**^, *p* < 0.01; ^***^, *p* < 0.001. (d–i) Effects of silencing the salicylic acid biosynthesis gene *GhICS1* and the salicylic acid receptor gene *GhNPR1* on cotton phenotype (d), *GhICS1* expression (e), *GhNPR1* expression (f), root SA content (g), root length colonization of AM fungi (h), and colonization structure of AM fungi (i). SA represents salicylic acid, CA represents chorismate, ICS represents isochorismate synthase, and ISC represents isochorismate. Two‐tailed Student's *t*‐test was used to assess statistical differences between two groups. Error bars indicate ±SD of independent replicates. WGA staining was used to visualize AM fungal colonization. Bar = 200 µm. Sample sizes were *n* = 9 for root length colonization, and *n* = 3 for gene expression analyses and SA content. ^*^, *p* < 0.05; ^**^, *p* < 0.01; ^***^, *p* < 0.001. (j–l) Effects of silencing the alternative salicylic acid biosynthesis gene *GhPAL10* on cotton phenotype (j), root SA content (k), and colonization structure of AM fungi (l). Phe, phenylalanine; PAL, phenylalanine ammonia‐lyase; t‐CA, *trans*‐cinnamic acid; SA, salicylic acid. Two‐tailed Student's *t*‐test was used to assess statistical differences between two groups. Error bars indicate ±SD of independent replicates. Sample sizes were *n* = 9 for root length colonization, and *n* = 3 for gene expression analyses and SA content. WGA staining was used to visualize AM fungal colonization. Bar = 200 µm. ^**^, *p* < 0.01; ^***^, *p* < 0.001.

Inoculation with AM fungi significantly inhibited the accumulation of SA in cotton roots but promoted the accumulation of JA and JA‐Ile (Figure 6a). In cotton, endogenous SA biosynthesis occurs through two pathways: the isochorismate (ICS) pathway and the phenylalanine ammonia‐lyase (PAL) pathway. AM fungal inoculation decreased the expression of SA biosynthesis genes *GhICS1* and *GhPAL10* (Figure S18a). Exogenous SA application reduced AM fungal colonization in cotton roots, accompanied by a decline arbuscule colonization (Figure 6b). Meanwhile, exogenous SA treatment upregulated the expression of pathogenesis‐related genes *GhPR1* and *GhPR3*, while downregulating the expression of the AM symbiosis marker gene *GhPT5* and the housekeeping gene of AM fungi *RiEF1α* (Figure 6c). Exogenous SA application decreased the expression of JA biosynthesis genes *GhAOS1* and *GhOPR3* but had no effect on the expression of SA biosynthesis genes *GhICS1* (Figure 6c and Figure S18b).

To investigate the regulatory mechanism of SA in AM symbiosis, two key SA biosynthesis genes (*GhICS1* and *GhPAL10*) were silenced (Figure 6d,e,j). Silencing of *GhICS1* or *GhPAL10* decreased endogenous SA levels (Figure 6g,k), accompanied by an increase in AM fungal colonization and arbuscule density in cotton roots (Figure 6h,l). Silencing of *GhICS1* downregulated the expression of pathogenesis‐related genes *GhPR1*, *GhPR3*, and *GhPR4* (Figure S19a). *GhPAL10* silencing dramatically upregulated AM symbiotic marker genes *GhPT5* and *GhHA1* (Figure S19b). However, changes in endogenous SA levels did not affect the development of arbuscule structures, and no abnormalities or premature senescence of arbuscules were observed (Figure 6i,j). GhNPR1 serves as the SA receptor in cotton. Silencing of *GhNPR1* decreased endogenous SA levels and increased AM symbiosis in cotton roots (Figure 6f,g,h). These results support that SA acts as a negative regulator of AM symbiosis in cotton.

### 2.7 *GhNTF3* and *GhWAK13* Exhibit Interdependent Regulation of AM Symbiosis

**FIGURE 7:**
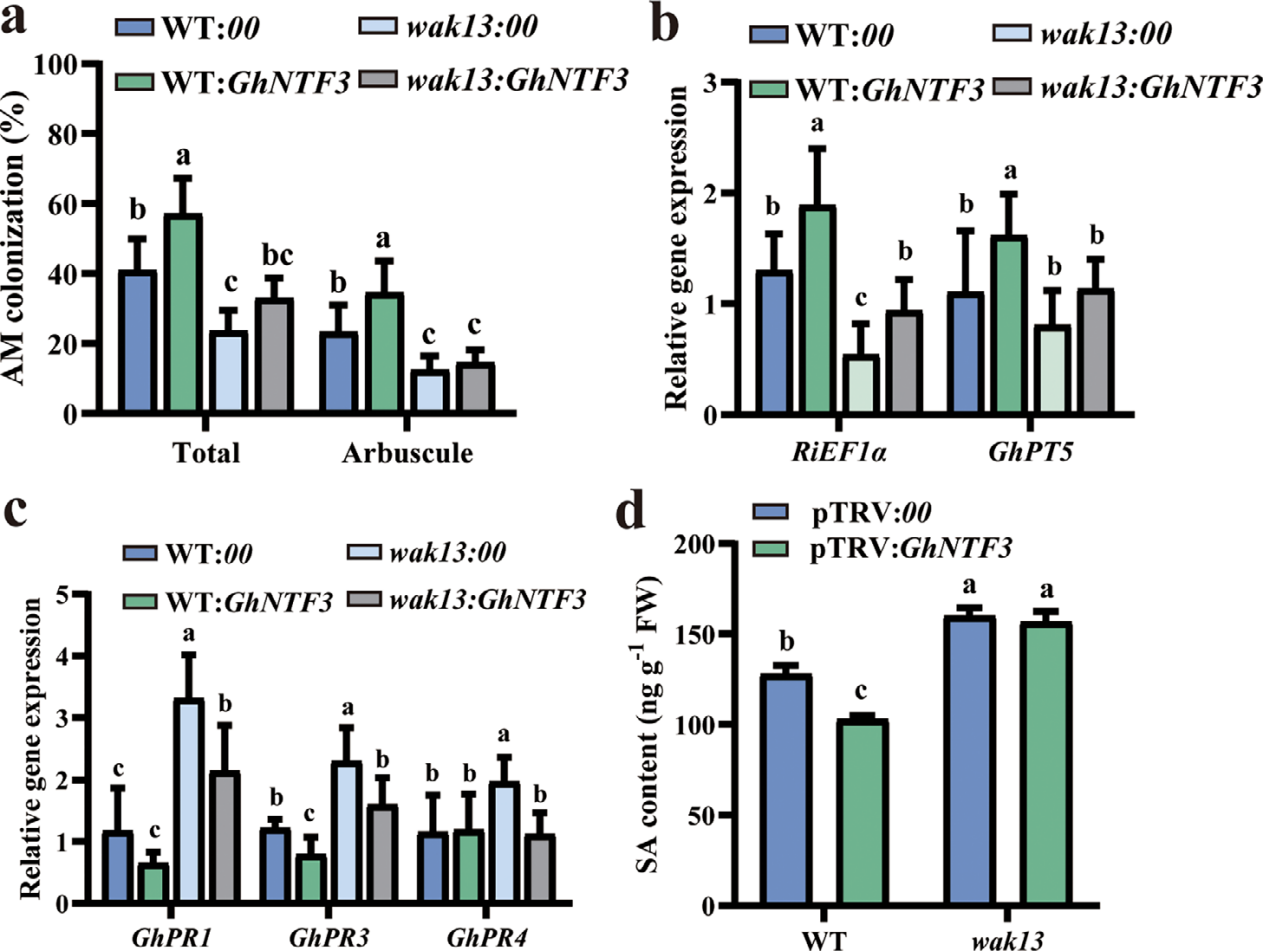
Genetic interaction analysis of *GhNTF3* and *GhWAK13* during AM symbiosis. (a) Total and arbuscule colonization in both *GhWAK13*‐knockout and *GhNTF3*‐silenced cottons. WT:*00* represents wild‐type cotton without *GhNTF3* silencing. *wak13*:*00* represents *GhWAK13*‐knockout cotton without *GhNTF3* silencing. WT:*GhNTF3* represents wild‐type cotton with *GhNTF3* silencing. *wak13*: *GhNTF3* represents *GhWAK13*‐knockout cotton with *GhNTF3* silencing. Error bars indicate ±SD of independent replicates. One‐way ANOVA was used to determine statistically significant differences among treatments, followed by Duncan‐test post hoc analysis. Each parameter was analyzed separately using ANOVA. Different letters indicate significant differences among treatments. *n* = 9. *p* < 0.05. (b, c) Effects of *GhNTF3* silencing and *GhWAK13* knockout on AM marker genes (b) and defense genes (c) in cottons. WT:*00* represents wild‐type cotton without *GhNTF3* silencing. *wak13*:*00* represents *GhWAK13*‐knockout cotton without *GhNTF3* silencing. WT:*GhNTF3* represents wild‐type cotton with *GhNTF3* silencing. *wak13*: *GhNTF3* represents *GhWAK13*‐knockout cotton with *GhNTF3* silencing. *GhUBQ7* was used as a housekeeping gene in cotton. Error bars indicate ±SD of independent replicates. One‐way ANOVA was used to determine statistically significant differences among treatments, followed by Duncan‐test post hoc analysis. *n* = 8. *p* < 0.05. (d) SA contents in cotton roots under *GhNTF3* silencing and *GhWAK13* knockout treatments. Error bars indicate ±SD of independent replicates. One‐way ANOVA was used to determine statistically significant differences among treatments, followed by Duncan‐test post hoc analysis. *n* = 3. *p* < 0.05.

*GhNTF3* and *GhWAK13* exhibit mutual transcriptional repression. Specifically, silencing of *GhNTF3* led to an increase in *GhWAK13* transcript levels in AM roots, whereas knockout of *GhWAK13* resulted in elevated *GhNTF3* expression (Figure S20). Genetic interaction analysis further showed that the regulation of AM symbiosis mediated by GhNTF3 and GhWAK13 is tightly interconnected. Silencing of *GhNTF3* in the *ghwak13* mutant background led to AM symbiosis phenotypes similar to those of the *ghwak13* mutant (Figure 7a). However, the AM fungal colonization in *GhNTF3*‐silenced plants within the *ghwak13* mutant background was significantly higher than that in the *ghwak13* mutant. These results were consistent with the expression patterns of AM symbiosis marker genes *GhPT5* and the housekeep gene of AM fungi *RiEF1α* (Figure 7b). In addition, the transcriptional levels of the SA marker gene *GhPR1/3* were upregulated by *GhWAK13* knockout with or without *GhNTF3* silencing (Figure 7c), which was consistent with SA content and SA biosynthesis gene expression in roots (Figure 7d and Figure S21). These results suggest that *GhWAK13* is closely associated with the regulatory pathway mediated by *GhNTF3* in regulating SA accumulation and AM symbiosis.

### 2.8 GhJAZ6 Positively Regulates Verticillium Wilt Resistance and Interacts With GhNTF3 in the Nucleus

**FIGURE 8:**
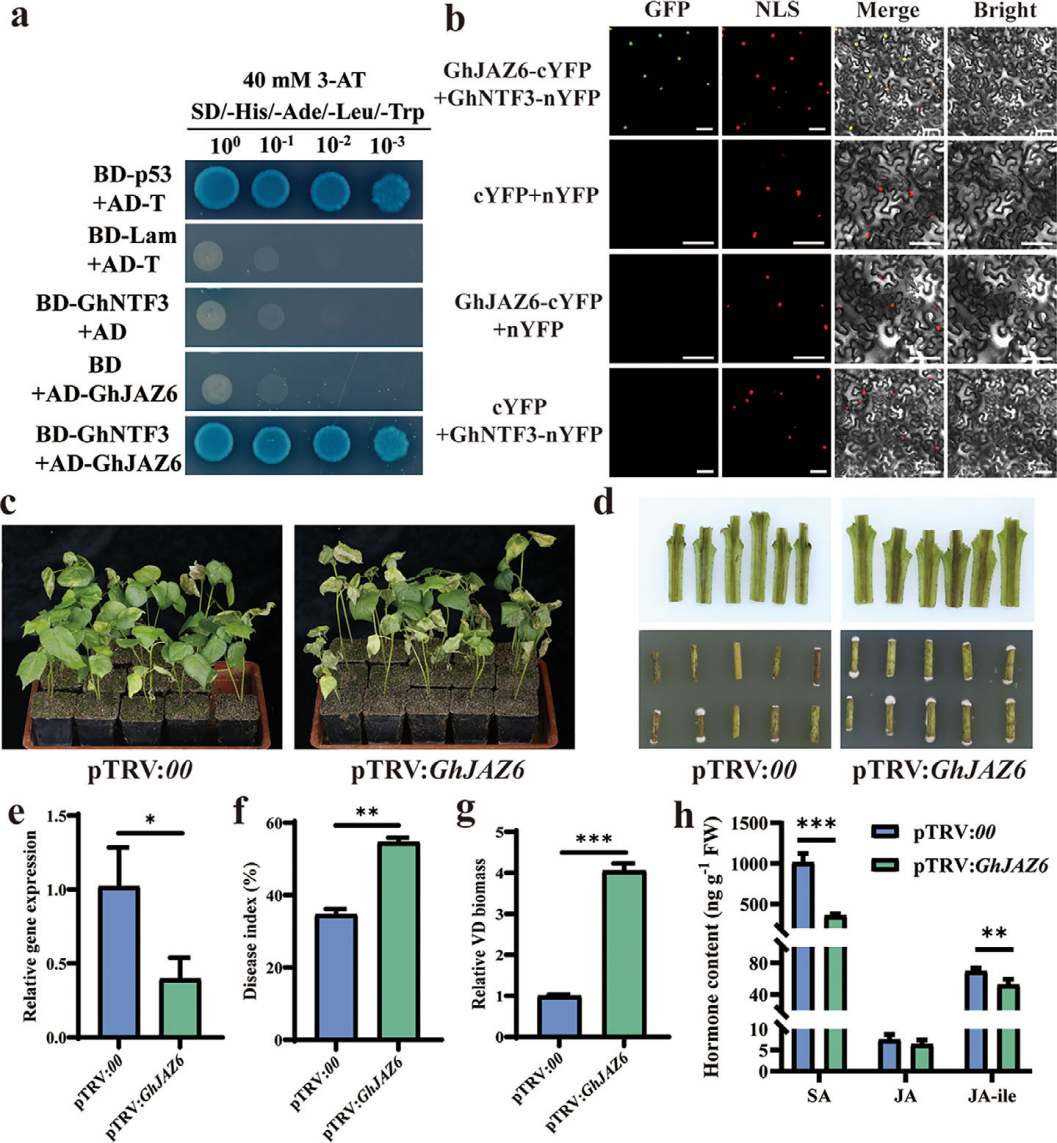
GhNTF3 interacts with GhJAZ6 in the nucleus. (a) GhNTF3 interacts with GhJAZ6 in the yeast two‐hybrid system. 40 mm 3‐AT was used to suppress false‐positive results. BD‐p53+AD‐T was used as a positive control. BD‐Lam+AM‐T was used as a negative control. (b) GhNTF3 interacts with GhJAZ6 in the nucleus. Bar = 50 µm. (c) The role of GhJAZ6 in Verticillium wilt resistance. At least 15 cotton seedlings were observed to determine the Verticillium wilt resistance. (d) The degree of vascular browning and stem fungal load in *GhJAZ6*‐knockdown cottons. (e–h) Effects of *GhJAZ6* on the relative expression of *GhJAZ6* (e), disease index (f), relative VD biomass (g), and hormone content (h) in cottons. SA, salicylic acid; JA, jasmonic acid; JA‐Ile, jasmonoyl‐isoleucine. Two‐tailed Student's *t*‐test was used to assess statistical differences between pTRV:*00* and pTRV:*GhJAZ6* plants. Error bars indicate ±SD of independent replicates. Sample sizes were *n* = 3 for gene expression analyses and disease index, and *n* = 4 for hormone content. ^*^, *p* < 0.05; ^**^, *p* < 0.01; ^***^, *p* < 0.001.

In the presence of 40 mm 3‐AT, which suppressed the autoactivation of GhJAZ6, a protein‐protein interaction between GhNTF3 and GhJAZ6 was confirmed (Figure 8a). The interaction between GhNTF3 and GhJAZ6 in the nucleus was further confirmed by a bimolecular fluorescence complementation assay (Figure 8b). Silencing of *GhJAZ6* decreased the Verticillium wilt resistance 2 weeks post *Vd991* infection (Figure 8c,e). *GhJAZ6‐s*ilencing resulted in a significant increase in disease severity, with leaves displaying enhanced yellowing and wilting compared with control cotton. In *GhJAZ6*‐silenced cotton, the degree of vascular browning was stronger than that in control cotton (Figure 8d). *GhJAZ6*‐silencing increased both stem fungal biomass (Figure 8d,g) and disease index (Figure 8f) compared with control cotton. *GhJAZ6* silencing decreased SA and JA‐Ile contents by 64.5% and 24.6%, respectively (Figure 8h). Silencing *GhJAZ6* significantly reduced the expression of the SA synthesis gene *GhPAL10*, but had little effect on *GhICS1* (Figure S22a). We also investigated the function of *GhJAZ6* on AM fungal symbiosis and found that silencing *GhJAZ6* promoted AM fungal root length colonization in cotton, which was consistent with the expression changes of the AM symbiosis marker genes *GhPT5* and *GhBCP1* (Figure S22b,c).

## 3 Discussion

### 3.1 The MAPK Protein GhNTF3 Functions as a Negative Regulator of AM Symbiosis

AM fungi established mutualistic relationships with plants approximately 450 million years ago [27]. Plants recruit AM symbiotic signaling via conserved genetic modules, while simultaneously maintaining defense mechanisms against pathogens. This dynamic balance drives the co‐evolution of plants and microbes [28, 29]. Root epidermal cells perceive AM fungal‐derived symbiotic signals, including chitotetraose (CO4) and lipo‐chitooligosaccharides (Myc‐LCOs), through plasma membrane‐located receptor kinase heterodimers (CERK1‐MYR1) and DOES NOT MAKE INFECTIONS 2 (DMI2). These recognition events transmit symbiotic signals via phosphorylation cascades to downstream factors, including cyclin‐dependent kinase‐like 1/2 (CKL1/2), subsequently mediating the expression of symbiotic‐related genes [21, 30, 31, 32]. Similarly, plants perceive pathogen‐derived chitin through receptor kinase heterodimers such as CERK1‐CEBiP or CERK1‐LYK5, thereby initiating phosphorylation‐dependent activation of downstream signaling cascades and inducing the expression of defense genes [11, 33]. These findings reveal phosphorylation‐dependent regulation as a key molecular switch in plant‐microbial interactions.

Although key components underlying AM symbiosis signaling have been gradually elucidated, the immune regulators coordinating this interaction remain poorly characterized. To address this gap, we performed phosphoproteomic profiling, which revealed extensive dephosphorylation events in the MAPK cascade, plant‐pathogen interaction, and plant hormone signal transduction during AM symbiosis (Figure 1), suggesting that immune signaling is dynamically reprogrammed to facilitate AM symbiosis. It is well known that MAPK signaling cascades serve as central regulators of plant immunity [34]. Further analysis indicates that MAPK cascades act as a core regulatory module in AM symbiosis. (Figures S3 and S4). Upon pathogen infection, MPK3/6 serve as signaling hubs in plant immunity by integrating upstream phosphorylation signals (e.g., MKK4/5, MAPKKK3/5) [35] and phosphorylating downstream components such as transcription factors WRKY18/33 [36], thereby regulating immune response. Our results showed that MPK3/6 were unaffected by AM fungal regulation, whereas a novel MAPK, GhNTF3, was essential for both AM symbiosis and immune responses (Figures 2 and 3), highlighting its potential role in coordinating the trade‐off between symbiosis and immunity. Protein domain and evolutionary analyses revealed that GhNTF3 is a member of the extracellular signal‐regulated kinase‐like (ERK‐like) family, characterized by a highly conserved threonine‐glutamate‐tyrosine (TEY) motif in the activation loop [17]. GhNTF3 has undergone lineage‐specific divergence, potentially driven by partial domain loss, leading to the formation of distinct clades separating AM symbiotic species from non‐AM symbiotic lineages such as Brassicaceae family (Figure 2a). This evolutionary divergence emphasizes the functional significance of GhNTF3 in modulating AM symbiosis.

In non‐AM *Arabidopsis thaliana*, MPK1/2 are homologs of GhNTF3. AtMPK1/2 is activated by wounding, such as insect herbivory, where they mediate innate immune response independently of the MPK3/6 signaling cascade [37]. In AM cotton, *GhNTF3* negatively regulates AM symbiosis by modulating arbuscule number and density in cortical cells without significantly affecting arbuscule development (Figure 2i), suggesting its potential role during the early stages of symbiosis. This result was further confirmed by the observation that *GhNTF3*‐overexpressing tobacco plants suppressed AM fungal colonization (Figure 2k). Stable CRISPR‐Cas9 knockout lines of *ghntf3* in cotton are currently being generated. Due to the long transformation time, they are not yet available for this study, and further analysis of *ghntf3* lines will further support our conclusions. Meanwhile, 94% of the *GhNTF3*‐downregulated gene clusters were significantly enriched in symbiosis‐related pathways (Figure S14), indicating that GhNTF3 functions as a repressor of AM fungal colonization. This conclusion is further supported by the enhanced growth observed in *GhNTF3*‐silenced cotton, in which AM symbiosis promotes plant performance by improving phosphate and nitrogen acquisition [38, 39]. Under biotic stress, MPK1/2 can be activated by MKK3 to transduce extracellular signals into intracellular defense responses, including the activation of nodule inception‐like protein (NLP) transcription factors [40]. Consistently, transcriptomic profiling revealed that 25% of *GhNTF3*‐upregulated gene clusters were enriched in *V. dahliae* infection response (Figure S14). These genes were predominantly associated with phenylpropanoid biosynthesis, a pathway essential for disease resistance. In Verticillium wilt‐resistant cotton, regulators of phenylpropanoid metabolism are activated by the transcription factor WRKY41, promoting lignin and flavonoid accumulation [41], which likely contributes to the enhanced resistance observed in *GhNTF3* plants. Collectively, these findings demonstrate that *GhNTF3* acts as a regulator that suppresses AM symbiosis while enhancing defense responses, thereby mediating the trade‐off between symbiotic and immunity.

### 3.2 GhNTF3‐GhWAK13 Module Acts as a Molecular Switch in the Trade‐Offs Between AM Symbiosis and Immunity

We have identified *GhNTF3* as a negative regulator of AM symbiosis. However, fine‐tuned regulation of AM symbiosis often relies on complex interactions among multiple factors. Recently, several signaling modules, including BAK1‐SmRK, CERK1‐LYK8, along with key genes such as LSM1 and WAK13, have been shown to be involved in symbiosis‐mediated immune suppression [2, 9, 24, 42, 43]. In non‐AM plants, *A. thaliana* employs FERONIA‐mediated suppression of PTI to facilitate colonization by phosphate‐mobilizing microbiota, thereby mitigating phosphate starve stress [44]. Yeast library screening revealed that GhNTF3 may interact with a range of proteins, including the membrane protein GhWAK13, nuclear proteins GhJAZ6 and GhWRKY41 (Table S1). MAPKs receive upstream signals in the cytoplasm and then translocate to the nucleus, where they modulates downstream gene transcription through interactions with transcription factors or regulatory proteins [26, 45]. This finding is consistent with the observed localization of GhNTF3 in both the nucleus and cytoplasm.

Wall‐associated kinases were previously considered receptors for oligogalacturonides (OGs), a well‐characterized class of DAMPs, due to its extracellular EGF domain in previous opinion [46]. Recently, accumulating evidence suggests that WAKs act as scaffold for pattern recognition receptor complexes, facilitating immune signaling and enhancing disease resistance [11, 47]. GhWAK7A is a homolog of GhWAK13. Upon *V. dahliae* infection, GhWAK7A forms a heterotetrameric complex with chitin recognition modules CERK1‐LYK5, thereby transmitting a defense signal to the nucleus via activation of the MAPK pathway in the cytoplasm [11]. In this study, GhWAK13, previously characterized as a positive regulator of AM symbiosis [2], showed genetically antagonistic interactions with GhNTF3 in the regulation AM symbiosis (Figure 2). Genetic analysis indicates that *GhWAK13* and *GhNTF3* do not follow a simple linear upstream/downstream relationship in the regulation of AM symbiosis. Although silencing of GhNTF3 in the *ghwak13* mutant background resulted in a phenotype generally similar to that of *ghwak13* mutant, AM fungal colonization in this line was still higher than in the *ghwak13* mutant. These results suggest that GhWAK13 plays a predominant role in AM symbiosis regulation, while GhNTF3 can modulate AM symbiosis through a GhWAK13‐independent pathway. This interpretation is further supported by phosphorylation data. Alteration of GhWAK13 activity affected the phosphorylation status of GhNTF3 (Figure S23), whereas GhWAK13 was unable to directly phosphorylate GhNTF3 in vitro. These findings suggest that GhWAK13 influences GhNTF3 activity through an indirect mechanism, potentially involving additional signaling components. Similarly, GbWAKL14, a WAK‐like protein, negatively regulates Verticillium wilt resistance by inhibiting phosphorylation of GbMPK3 to reduce the defense signal transducing into the nucleus with the help of protein phosphatase 2C [26]. In tomato, SiWAK1 act as a scaffold rather than a kinase, promoting interactions between SiFls2 and SiFls3 to trigger immune responses against *Pseudomonas syringae* [47].

Therefore, we propose that mutual regulation between GhWAK13 and GhNTF3 may be influenced by the subcellular localization of GhNTF3. The phosphorylation status of GhNTF3 was altered by changes in GhWAK13 abundance during AM fungal colonization (Figure S23). GhNTF3 phosphorylation was reduced in the *ghwak13* mutant and increased in *GhWAK13*‐overexpression plants, indicating that GhWAK13 is required to maintaining normal GhNTF3 phosphorylation. The increased phosphorylation of GhNTF3 observed in *GhWAK13*‐overexpressing plants may reflect prolonged residence of GhNTF3 within membrane‐associated signaling complexes, where it remains accessible to other kinases. Because GhWAK13 did not directly phosphorylate GhNTF3 in vitro, its effect is likely indirect. GhWAK13 physically interacts with GhNTF3 at the plasma membrane, and subcellular fractionation further suggests that GhWAK13 might affect the subcellular distribution of GhNTF3 (Figure S24). In the presence of GhWAK13, a larger proportion of GhNTF3 was retained outside the nucleus, whereas loss of GhWAK13 promoted the accumulation of GhNTF3 in the nucleus. These observations suggest that GhWAK13 influences not only phosphorylation status but also intracellular localization of GhNTF3. GhWAK13 may suppress the nuclear translocation of GhNTF3‐mediated immune signals, thereby blocking downstream immune activation.

In addition, interaction between the kinase domains of GhWAK13 and GhNTF3 may competitively inhibit the binding of other proteins, leading to their functional antagonism. Similarly, WAKL20 interacts with Barley Stripe Resistance 1 (BSR1) to inhibits BSR1‐mediated immunity via steric hindrance within the WAKL20‐BSR1 complex to suppresses BSR1‐mediated downstream immune signaling [48]. All in all, *GhNTF3* maintains innate immunity through its constitutive expression. During AM symbiosis, induced *GhWAK13* expression facilitates formation of the GhNTF3‐GhWAK13 complex, suppressing NTF3‐mediated immune responses to establish a suitable symbiotic environment. Conversely, upon pathogen infection, suppressed *GhWAK13* expression releases *GhNTF3*‐mediated immunity, simultaneously disrupting the AM symbiotic environment and enhancing disease resistance. The GhNTF3‐GhWAK13 module functions as a molecular switch balancing AM symbiosis and immunity to maintain cotton normal growth.

### 3.3 Salicylic Acid Contributes to *GhNTF3*‐Mediated Regulation of AM Symbiosis

Phytohormones play pivotal roles in plant physiological processes, particularly in plant immunity. Among them, SA is a key defense hormone regulating resistance against biotrophic pathogens such as bacteria [21]. SA serves as a central regulator of systemic acquired resistance (SAR) and coordinates defense‐related processes such as the hypersensitive response [49], which may antagonize AM symbiosis. This was supported by the potential negatively effect of SA on AM fungi symbiosis relationship [50, 51, 52, 53]. However, direct evidence for the role of SA in AM fungal symbiosis remains limited. GhICS1 and GhPAL10 function as key enzymes for SA biosynthesis via the isochorismate and phenylpropanoid pathways, respectively [54, 55]. In this study, both exogenous SA application and inhibition of endogenous SA biosynthesis significantly disrupted AM symbiosis in cotton, demonstrating that SA acts as a negative regulator of AM fungal colonization. NPR1 is the central SA receptor responsible for downstream immune signaling [56]. Silencing of *GhNPR1* promoted AM symbiosis, accompanied by decreased SA levels (Figure 6), further supporting this point. However, neither SA biosynthesis impairment nor *GhNPR1* knockdown altered arbuscule development, indicating that SA may regulate the pre‐symbiotic phase [51] rather than post‐penetration symbiotic development.

Previous study reported that MAPK protein MEK1 promotes SA accumulation and mediates SA‐dependent leaf senescence [57]. Our metabolomic analysis showed that *GhNTF3* is associated with increased SA accumulation in cotton roots, whereas *GhWAK13* shows an opposite regulatory pattern. This is consistent with their contrasting effects on AM symbiosis (Figure 5). In line with SA levels, *PR* gene expression exhibited similar trends. Exogenous SA treatment further suggests that SA is involved in the regulation of AM symbiosis mediated by *GhNTF3* and *GhWAK13*, although its role still needs further clarification. Recent work identified ARK as a conserved regulator required for maintaining AM symbiosis across land plants [58]. Unlike ARK, which mainly supports symbiotic maintenance, GhNTF3 appears to affect the outcome of AM symbiosis by modulating host immune responses. These findings indicate that AM symbiosis is regulated by both conserved symbiotic pathways and immunity. Genetic analysis further suggests that *GhNTF3* and *GhWAK13* are functionally interconnected during AM symbiosis rather than in a strict linear regulatory hierarchy. *GhWAK13* shows a closer association with symbiotic phenotypes compared with *GhNTF3*, which is consistent with the observed differences in SA accumulation (Figure 7). We propose that GhWAK13 may influence the subcellular behavior of GhNTF3 in AM roots, thereby affecting its regulatory function. In addition, GhWAK13 may regulate an unidentified factor involved in SA biosynthesis, which requires further investigation. Taken together, our results support a model in which GhNTF3 and GhWAK13 are associated with AM symbiosis through differential regulation of SA‐related immune responses, rather than through a simple linear signaling pathway.

The decreased SA in AM roots was accompanied by increased levels of JA, JA‐Ile, and *trans*‐zeatin (TZ), while the content of 1‐aminocyclopropane‐1‐carboxylic acid (ACC) and indole‐3‐acetic acid (IAA) were deceased, consistent with the change in SA (Figure 5c). These changes indicate that the effects of *GhNTF3* are not restricted to SA and involve a broader hormonal reprogramming. JA plays a central role in plant immunity, primarily mediating fungal pathogen or insect resistance [20]. Jasmonic acid has been reported to either promote [59] or suppress mycorrhizal symbiosis [60, 61], and these contrasting effects may depend on its concentration. The accumulation of JA and JA‐Ile in *GhNTF3*‐silenced roots suggests that JA may contribute to reduced AM colonization. However, *GhNTF3* showed an opposite regulation pattern under *V. dahliae* infection (Figure 3g), suggesting that its effect on JA is likely indirect. Verticillium wilt resistance depends on the regulation of the JA and SA signaling pathway [62, 63]. Our study also proves that *GhNTF3* positively regulates Verticillium wilt resistance via enhancing JA accumulation in roots (Figure 3). Similarly, MPK1/2 are reported to positively regulate the biosynthesis of JA to resist biotic stress [37]. In strawberry, MPK6 interacts with JAZ12 to attenuate its nuclear accumulation, thereby suppresses JA signaling [64]. Our results reveal that GhNTF3 physically interacts with GhJAZ6 in the nucleus (Figure 8). Interesting, GhJAZ6 in cotton shows a regulatory pattern that differs from canonical JAZ proteins [65, 66]. In the canonical JA signaling pathway, JAZ proteins act as transcriptional repressors in a COI1‐dependent manner [67]. However, our results suggest that GhNTF3 and GhJAZ6 may form a regulatory complex that is involved in hormone regulation beyond the classical JA signal pathway. The decrease of JA‐Ile observed in *GhJAZ6*‐silenced plants indicates that GhJAZ6 may not function only as a negative regulator in JA signaling. One possible explanation is that the GhNTF3‐GhJAZ6 complex may be involved in regulating upstream components related to JA metabolism, thereby affecting JA‐Ile accumulation. This could lead to changes in hormone homeostasis rather than a simple alteration in canonical JA signaling pathway [68]. In addition, the changes in SA levels suggest that this regulatory module may participate in the coordination between JA‐ and SA‐related pathways during AM symbiosis. Consistent with this, the phenotype of *GhJAZ6*‐silenced plants further supports its role in hormone regulation, although the detailed molecular mechanism still needs further investigation. TZ is a kind of cytokinin that has been reported to positively regulate AM symbiosis [69]. ACC is the immediate precursor of ethylene in plants and act as a negative regulator of AM symbiosis [70]. In this study, *GhNTF3* silencing led to increased TZ levels but decreased ACC levels, which is consistent with the enhanced AM fungal colonization in cotton. These findings provide additional evidence supporting the hypothesis that *GhNTF3* enhances Verticillium wilt resistance and suppresss AM fungal symbiosis by modulating multiple hormone pathways.

**FIGURE 9:**
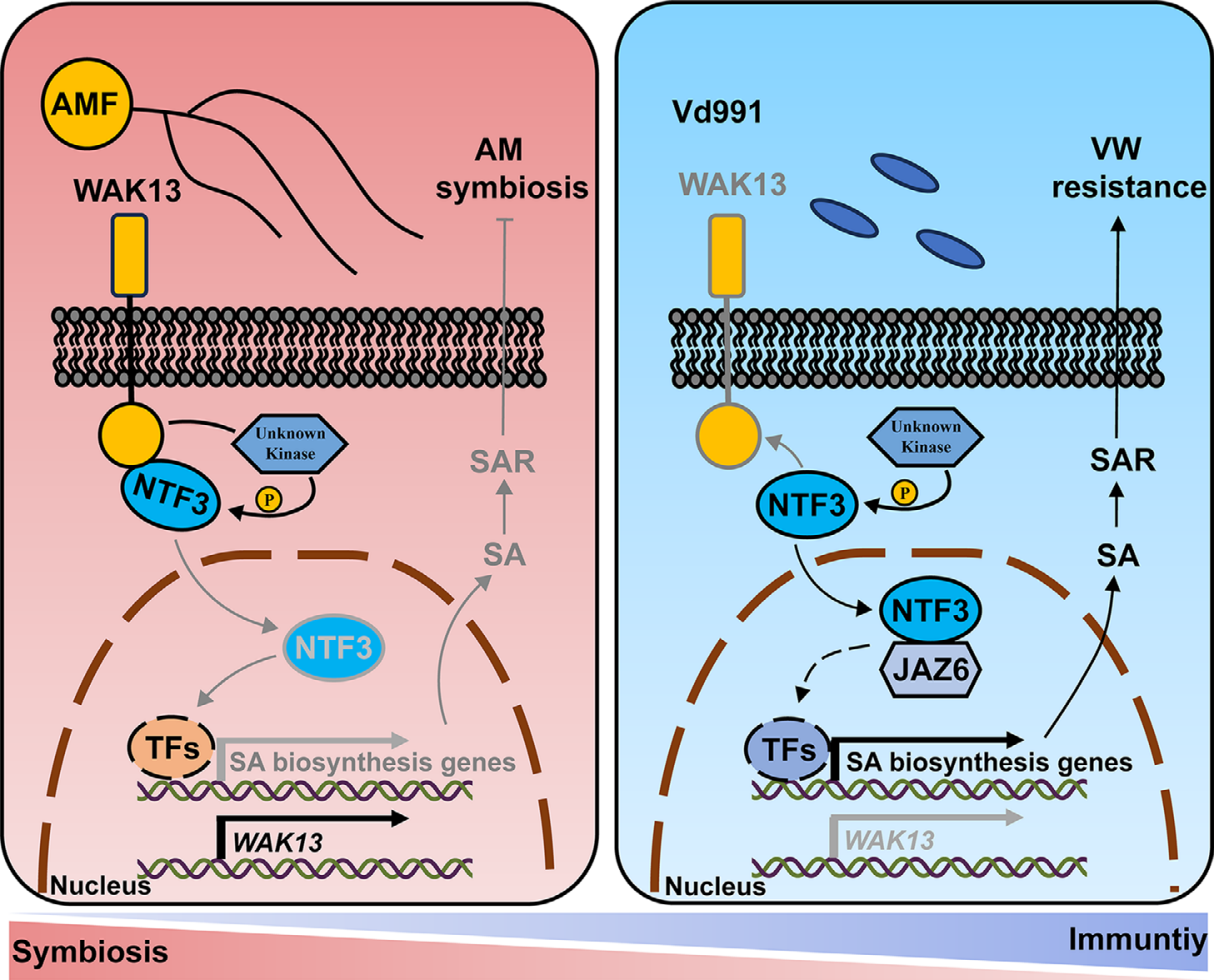
Hypothetical model of GhNTF3 mediated regulation of AM symbiosis and immunity balance. In brief, AM symbiosis induces the expression of the cell wall‐associated kinase GhWAK13, which is localized to the periarbuscular membrane (PAM) and positively regulates AM fungal colonization. GhNTF3 functions as a negative regulator of AM symbiosis and is involved in the regulation of salicylic acid (SA) biosynthesis, thereby preventing excessive AM fungal colonization. During AM symbiosis, GhWAK13 is proposed to modulate the subcellular localization of GhNTF3 at the PAM, which may restrict its nuclear accumulation and consequently attenuate SA‐associated suppression of AM symbiosis. Under Verticillium wilt stress, GhNTF3 shows increased nuclear accumulation and is associated with the activation of SA‐ and JA‐related defense responses, potentially through interactions with components of hormone signaling pathways. The GhNTF3 module functions as a molecular switch regulating symbiosis‐immunity trade‐offs.

In conclusion, we identified a novel MAPK protein, GhNTF3, through phosphoproteomic analysis. GhNTF3 negatively regulates AM symbiosis while positively modulating resistance to Verticillium wilt in cotton. GhNTF3 is localized in both the cytoplasm and nucleus. It is associated with the wall‐associated kinase GhWAK13 at the plasma membrane. This is accompanied by reduced nuclear accumulation of GhNTF3 during AM symbiosis. GhNTF3 is accompanied by elevated SA levels in AM roots. In contrast, GhWAK13 shows an opposite trend and may help maintain SA homeostasis during symbiosis. Under Verticillium wilt infection, GhNTF3 accumulates in the nucleus. It interacts with GhJAZ6, coinciding with activation of SA‐ and JA‐related defense responses. Collectively, our results suggest that GhNTF3 acts as a regulatory node coordinating the balance between AM symbiosis and immune responses in cotton, with SA‐related signaling pathways contributing to this process (Figure 9).

## 4 Materials and Methods

### 4.1 Plant Materials and Growth Conditions

The upland cotton (*Gossypium hirsutum* L.) wildtype used for virus‐induced gene silence (VIGS) were in the Texas Male Sterile‐1 (TM‐1) background. The upland cotton wildtype and gene‐edited plants used in this study were in the Jin668 background. Cotton seeds were chemically decorticated (98% H_2_SO_4_, 10 min), surface‐sterilized (5% NaClO, 15 min), and germinated on sterile moist gauze under a 14 h light at 28°C and 10 h dark at 24°C for 3 d. Plants were cultivated in mesh‐bottomed pots (300 g substrate) under controlled conditions (16/8 h light/dark, 28/23°C). For AM fungal symbiosis studies, a sterile sand: vermiculite (1:1 v/v) substrate was inoculated with 20 g of *Rhizophagus irregularis* culture (30 spores/g) containing colonized root fragments. Control treatments received autoclaved inoculum. All plants received weekly applications of low phosphate Hoagland solution (30 µm H_2_PO_4_^−^, pH 6.0) to maintain AM symbiosis. For Verticillium wilt infection, V991 strain microsclerotia were propagated in potato dextrose agar (PDA) medium at 28°C (120 rpm, 5 d), with conidial suspensions (1 × 10^7 spores/mL) prepared for root‐dip inoculation. Plants were dark‐incubated (48 h) before resuming normal photoperiod post infection.

### 4.2 AM Fungal Colonization Assessment and Microscopic Analysis

Root samples were processed for AM fungal structure visualization using dual staining approaches. For bright field microscopy, roots were washed free of soil, sectioned into 1 cm segments, and sequentially treated with: (1) 5% KOH at 90°C for 30 min for tissue clearing, (2) 1% HCl at room temperature for 5 min for pH neutralization, and (3) 0.05% (w/v) trypan blue in lactic acid at 90°C for 30 min for fungal staining. After destaining in 1:1 (v/v) lactic acid: glycerol at 90°C for 30 min, samples were examined under a Zeiss Axio Imager A2 microscope. Colonization rates were quantified following McGonigle et al. [71]. For fluorescence detection, roots underwent ethanol dehydration (50%, 5 h), alkaline clearing (20% KOH, 48–72 h), acidification (0.1 m HCl, 20 min), and phosphate buffered solution washes before overnight staining with 1 µg·mL^−^^1^ WGA‐Alexa Fluor 488 (Invitrogen). Fluorescent signals were captured using a Nikon A1 SIM confocal system (excitation/emission: 488/500–550 nm).

### 4.3 Phosphoproteomic Profiling Assay

Protein extracts were quantified by BCA assay and quality‐checked via SDS‐PAGE prior to tryptic digestion (1:50 enzyme‐to‐protein ratio, 16 h at 37°C) following disulfide bond reduction and cysteine alkylation. Desalted peptides were subjected to phosphoenrichment using TiO_2_ microcolumns, then analyzed by nanoLC‐MS/MS (Q‐Exactive HF‐X) with a 90‐min acetonitrile gradient (5%–35%) on a reversed‐phase column (75 µm × 25 cm). Data‐independent acquisition (DIA) raw files were processed using Spectronaut for phosphosite identification, with differential phosphorylation defined as ≥ 2‐fold change, *p* < 0.05.

### 4.4 Virus‐Induced Gene Silencing Assay

Gene‐specific fragments were PCR‐amplified from cotton cDNA and cloned into the pTRV2 vector for sequence‐verified constructs. Recombinant plasmids were transformed into *Agrobacterium tumefaciens* GV3101 through heat shock. Bacterial cultures containing pTRV1, pTRV2 (empty vector control), pTRV2:CLA1 (positive control), and pTRV2:target‐gene were grown in induction medium (LB with 10 mm MES [pH 5.6], 20 µm acetosyringone, and appropriate antibiotics) at 28°C until OD600 reached 0.8–1.0. After centrifugation, bacterial pellets were resuspended in infiltration buffer (OD600 = 1.0) and incubated for 3 h at room temperature. For VIGS inoculation, pTRV1 cultures were mixed 1:1 (v/v) with pTRV2 derivatives and pressure‐infiltrated into cotyledons of two‐week‐old cotton seedlings. Systemic gene silencing was confirmed by photobleaching in pTRV2:CLA1 controls. Root tissues were harvested at the onset of phenotypic symptoms, flash‐frozen in liquid nitrogen, and stored at −80°C for downstream transcript analysis.

### 4.5 Total RNA Extraction From Cotton Tissues

Total RNA was isolated from cotton samples using a specialized plant RNA extraction kit (TIANGEN, Polysaccharides & Polyphenolics‐rich Plant Total RNA Kit) according to the supplier's protocol. To eliminate genomic DNA interference, an on‐column DNase digestion step was included. RNA integrity was verified by electrophoresis on a 1.2% denaturing agarose gel, and spectrophotometric analysis (NanoDrop 2000c, Thermo Fisher Scientific) was employed to assess RNA purity (A260/A280 and A260/A230 ratios) and quantify nucleic acid concentrations.

### 4.6 RNA‐seq Assay

Root samples from AM‐inoculated, non‐mycorrhizal (NM), *GhWAK13* mutant, and *GhNTF3*‐silenced cotton plants were harvested 5 w post‐treatment. Samples were stored at −80°C for further experiments. For *V. dahliae* (VD) infection assays, roots were collected 12 h post‐inoculation. RNA sequencing (Illumina NovaSeq 6000) was performed by Guangzhou Gene Denovo Biotechnology, with raw reads processed using Trimmomatic and aligned to the G. hirsutum TM‐1 genome (v2.1) via STAR. Differentially expressed genes (DEGs) were identified using DESeq2 with thresholds of ∣Fold change∣≥2 (AM/VD) or ∣Fold change∣≥1.5 (GhWAK13/GhNTF3i) at *p* < 0.05. Functional enrichment analysis (GO and KEGG pathway) was conducted using OmicShare Tools, with significance set at FDR<0.05.

### 4.7 Quantitative Reverse Transcription PCR (qRT‐PCR) Analysis

Complementary DNA (cDNA) synthesis was performed using a Novoprotein Scientific reverse transcription system (Suzhou, China). Subsequent qPCR amplification was conducted with a SYBR Green master mix (Novoprotein Scientific) on a real‐time thermal cycler. Transcript abundance was normalized against the stably expressed endogenous control gene *GhUBQ7* (GenBank: DQ116441) and calculated via the comparative threshold cycle (2^−ΔΔCT^) method. Data reproducibility was confirmed across three technical replicates, and statistical significance was evaluated using ANOVA in SPSS (v26.0) with post‐hoc Tukey tests (*p* < 0.05). All primers used in this experiment were shown in Dataset S2.

### 4.8 Phytohormone Content Assay

Samples stored at −80°C were ground into a fine powder in liquid nitrogen. Approximately 0.1 g of fresh weight sample was taken and added to 1 mL of extraction solution (methanol:water:formic acid, v:v = 80:19:1). The mixture was subjected to extraction by shaking overnight at 4°C (alternatively, shake vigorously for half an hour followed by overnight incubation at 4°C). The extract was then centrifuged at 4°C and 10 000 rpm for 15 min, and 800 µL of the supernatant was collected. This supernatant was dried using a nitrogen evaporator. The dried residue was redissolved in 100 µL of 40% methanol by thorough vortexing until completely mixed. After a brief centrifugation, the solution was filtered through a 0.22 µm organic membrane filter. The filtrate was transferred into a small vial with an insert, and the vial was sealed tightly with a pre‐notched cap. These prepared samples were then submitted to the Large Instrument Usage Platform at Henan University for phytohormone measurement using UPLC‐MS/MS.

### 4.9 Widely Targeted Metabolomics Assay

Root tissues from AM‐colonized *GhWAK13* mutant cotton (5‐week post‐inoculation) and corresponding controls were collected, immediately frozen in liquid nitrogen, and maintained at −80°C until analysis. Metabolite extraction and quantification were performed using ultrahigh performance liquid chromatography‐tandem mass spectrometry (UHPLC‐MS/MS, AB SCIEX QTRAP 6500 platform) with electrospray ionization in both positive and negative modes. A comprehensive plant metabolome database (> 10 000 annotated compounds) was employed for metabolite identification. Data processing included normalization, batch correction, and quality assessment using internal standards. Statistically significant metabolites were determined through orthogonal projections to latent structures‐discriminant analysis (OPLS‐DA, VIP score ≥1.0) combined with univariate (*t*‐test, *p* < 0.05), with a minimum fold‐change threshold of 1.5.

### 4.10 Phylogenetic Tree Construction

Protein sequences of GhNTF3 and its homologs were retrieved from COTTONOMICS (http://cotton.zju.edu.cn/) and NCBI (https://www.ncbi.nlm.nih.gov/) protein databases using BLASTP Multiple sequence alignment was performed using Clustal X, followed by phylogenetic tree construction via the Neighbor‐Joining (NJ) method in MEGA software. Bootstrap values were generated with 1000 replicates to assess branch support. The resulting phylogenetic tree file was exported from MEGA‐X and further visualized using the iTOL online tool (https://itol.embl.de/) for customized branch coloring and taxonomic labeling.

### 4.11 Subcellular Localization Assay

The complete coding sequence of GhNTF3 (stop codon excluded) was PCR‐amplified and fused to GFP under the control of the CaMV *35S* promoter (*35Spro*:*GhNTF3‐GFP*). For transient expression assays, Agrobacterium tumefaciens GV3101 strains harboring either *35Spro*:*GFP* (negative control) or *35Spro:GhNTF3‐GFP* constructs were cultured in antibiotic‐supplemented LB medium (OD600 = 0.8‐1.0). Bacterial cells were pelleted and resuspended in MMA buffer (10 mm MgCl_2_, 10 mm MES, 100 µm acetosyringone, pH 5.6) for tissue infiltration. Tobacco leaves were pressure‐infiltrated using needless syringes, while cotton roots were transformed via Agrobacterium co‐cultivation. After 48–72 h incubation, fluorescent signals were visualized using a Nikon A1R confocal microscope with the following settings: GFP excitation 488 nm/emission 500–550 nm; mCherry excitation 515 nm/emission 580–630 nm. All observations were conducted with appropriate controls to verify specific subcellular localization patterns.

### 4.12 Yeast Two Hybrid Assay

The yeast two‐hybrid assay was performed using NMY51 strain with pTSU2‐APP/pPR3‐N (negative control) and pTSU2‐APP/pNubG‐Fe65 (positive control) pairs. Yeast cultures were grown in YPDA medium at 30°C with shaking (230 rpm) until OD546 reached 0.6–0.8. For transformation, 200 ng of each plasmid was mixed with competent cells in PEG/LiAc solution (300 µL), incubated at 42°C for 45 min, then plated on selective media after resuspension in 0.9% NaCl. The primary library contained approximately 1.6*10^7^ cfu, with an average insert size of 1000 bp as verified by PCR. A total of approximately 5*10^5^ colonies were screened. Transformants were cultured at 30°C for 4 days before analysis.

### 4.13 GST‐Pull Down Assay

Purified fusion proteins, such as GST‐GhWAK13KD and His‐GhNTF3, or other experimental protein combinations, were mixed in equal amounts in 2 mL centrifuge tubes. Binding buffer consisting of 1× PBS with 0.1% NP‐40 was added, and the mixture was incubated on a silent mixer at 4°C for 2 h. Pre‐equilibrated Glutathione Beads (SA008005, Tiandirenhe) with 1× PBS were then added to the tubes, and the incubation continued for an additional 4 h at 4°C on the silent mixer. Following this incubation, the samples were centrifuged at 4°C and 500 g for 5 min, and the supernatant was discarded. The beads were washed six to eight times with 1 mL of 1× PBS, with each wash involving centrifugation at 4°C and 500 g for 5 min followed by removal of the supernatant. After washing, the beads were resuspended in an appropriate volume of 1× PBS, and an equal volume of protein loading buffer was added to mix thoroughly with the samples. The mixture was heated at 100°C for 10 min for denaturation, then centrifuged at room temperature at 13 000 rpm for 2 min before being analyzed by Western‐blot.

### 4.14 Bimolecular Fluorescence Complementation Assay

The full‐length coding sequences of *GhWAK13* and *GhNTF3* were cloned into the vectors pXY104‐cYFP (to create GhWAK13‐cYFP) and pXY106‐nYFP (to create nYFP‐GhNTF3), respectively. The resulting recombinant plasmids were then transformed into *A. tumefaciens* strain GV3101. The Agrobacterium cultures containing GhWAK13‐cYFP, nYFP‐GhNTF3, and mCherry were grown to an OD600 of 0.8–1.0, then centrifuged to collect the bacterial pellets, which were subsequently resuspended in infiltration buffer. The resuspended bacterial suspensions were co‐infiltrated into leaves of tobacco. Approximately 48–72 h post‐infiltration, leaf discs of 1 cm × 1 cm near the infiltration sites were collected. YFP fluorescence expression in the epidermal cells of these leaves was observed and imaged under a confocal laser scanning microscope.

### 4.15 Generation of *GhWAK13* CRISPR/Cas9 Knockout Cotton

*GhWAK13* knockout lines were generated in the cotton cultivar Jin668 using a CRISPR/Cas9‐mediated genome editing system [72]. Single‐guide RNAs (*sgRNAs*) targeting GhWAK13 were designed using the CRISPR‐P2.0 online tool (http://cbi.hzau.edu.cn/cgi‐bin/CRISPR) [73]. Two *sgRNA* target sites were incorporated into each CRISPR/Cas9 construct. Genetic transformation was performed using hypocotyl explants as recipient tissues following the Agrobacterium‐mediated transformation protocol described by Wang et al. [74]. Transgenic plants were selected on medium containing kanamycin. Mutations at the target sites were identified and genotyped in subsequent generations using the Hi‐TOM high‐throughput mutation detection platform [75]. Independent homozygous mutant lines were selected for further analyses.

### 4.16 Statistical Analysis

All statistical analyses were performed using SPSS statistics version 26.0 (IBM, USA). Graphical representations were generated using GraphPad Prism 10.0 (GraphPad Software, USA). Data are presented as mean ± standard deviation (SD). All experiments included biological replicates as indicated in the corresponding figure legends, and the sample number (n) for each experiment is specified therein. Data distributions were assessed for normality using the Shapiro‐Wilk test, and homogeneity of variance was evaluated using Levene's test. For comparisons between two groups, statistical significance was assessed using a two‐tailed Student's *t*‐test. For comparisons involving three or more groups, one‐way analysis of variance (ANOVA) was performed, followed by Duncan‐test post hoc analysis. *p* < 0.05. Transcriptomic data were analyzed using the OmicShare platform (https://www.omicshare.com), and metabolomic data analysis was performed using the Metware Cloud platform (https://www.metware.cn) according to the respective standard pipelines provided by the platforms.

## Author Contributions

**Xiangyu Zhang**, S.J., and **Xiao Zhang** designed research; S.J., J.W., J.L., M.F., Q.L., R.L., and Y.H., performed research; J.Z. and K.C. contributed new reagents/analytic tools; S.J. and Xiangyu Zhang analyzed data; and Xiangyu Zhang, S.J., and Xiao Zhang wrote the paper.

## Conflicts of Interest

The authors declare no conflicts of interest.

## Supporting information




**Supporting File 1**: advs77315‐sup‐0001‐SuppMat.pdf.

**Supporting File 2**: advs77315‐sup‐0002‐S1.xlsx.

**Supporting File 3**: advs77315‐sup‐0003‐S2.xlsx.

**Supporting File 4**: advs77315‐sup‐0004‐S3.xlsx.

## Data Availability

The raw data that support the findings of this study are available on Supporting Information Dataset S3 and Figure S25‐S26. The RNA sequencing data generated in this study have been deposited in the NCBI Sequence Read Archive (SRA) database under accession number PRJNA1497369.
