# Supplementary material for: The Mitogen‐Activated Protein Kinase GhNTF3 Modulates the Arbuscular Mycorrhizal Symbiosis‐Immunity Trade‐Off in Cotton by Regulating Salicylic Acid Biosynthesis
Source: Adv Sci (Weinh). 2026 Aug 25:e77315. Online ahead of print. doi: 10.1002/advs.77315 (PMC13505469; doi:10.1002/advs.77315)
Supplement: Supplementary file 1 [file ADVS-9999-e77315-s003.pdf]

Supporting Information for

The mitogen-activated protein kinase GhNTF3 modulates the arbuscular mycorrhizal symbiosis-immunity trade-off in cotton by regulating salicylic acid biosynthesis

Shuangjie Jia[a, c, 1], Jingshang Wen[a, 1], Jihang Li[a, 1], Meina Feng[a], Yiming He[a], Ruojun Liu[a], Qian Liu[a], Junli Zhang[a], Kai Cheng[a], Xiangyu Zhang[a, 2], Xiao Zhang[a, b, 2]

[a] State Key Laboratory of Cotton Bio-breeding and Integrated Utilization, School of Life Sciences, Henan University, Kaifeng, 475001, China

[b] College of Life Sciences, Henan Agricultural University, Zhengzhou, 450002, China

[c] Institute of Edible Fungi, Henan Academy of Agricultural Sciences, Zhengzhou 450002, Henan, China

[1] These authors contributed equally to this work

[2] Corresponding authors: Xiangyu Zhang: xiangyuzhang0588@henu.edu.cn; Xiao Zhang: xzhang@henu.edu.cn

This PDF file includes:

Supporting text
Figures S1 to S26
Table S1
Legends for Datasets S1 to S3

Other supporting materials for this manuscript include the following:

Datasets S1 to S3

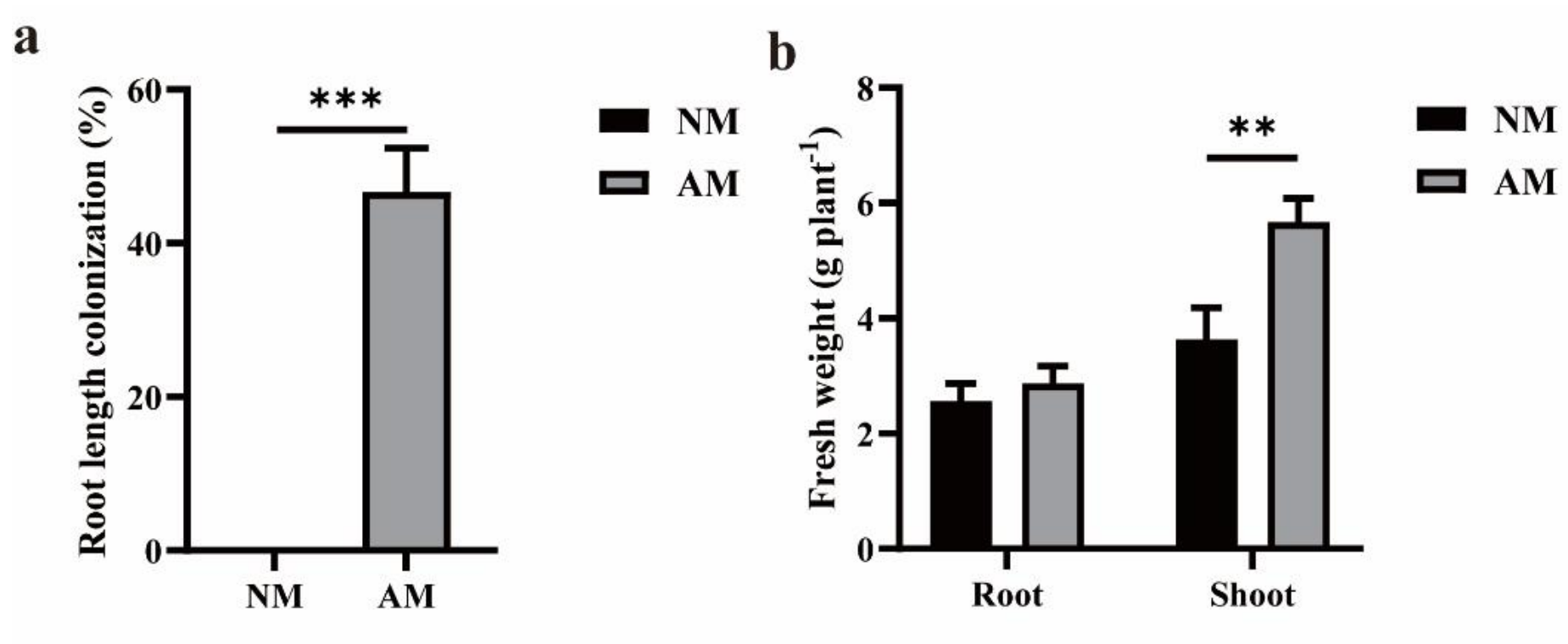

Figure S1. Effects of AM fungal inoculation on cotton growth. (a) The root length colonization in cotton roots 5 weeks post inoculation. n=3. Error bars indicate ±SD of independent replicates. Two-tailed Student's *t*-test was used to analyze the difference between nonmycorrhizal (NM) and AM plants. **, p< 0.01. (b) Fresh weight of mycorrhizal cottons. n=3. Error bars indicate ±SD of independent replicates. Two-tailed Student's *t*-test was used to analyze the difference between NM and AM plants. ***, p< 0.001.

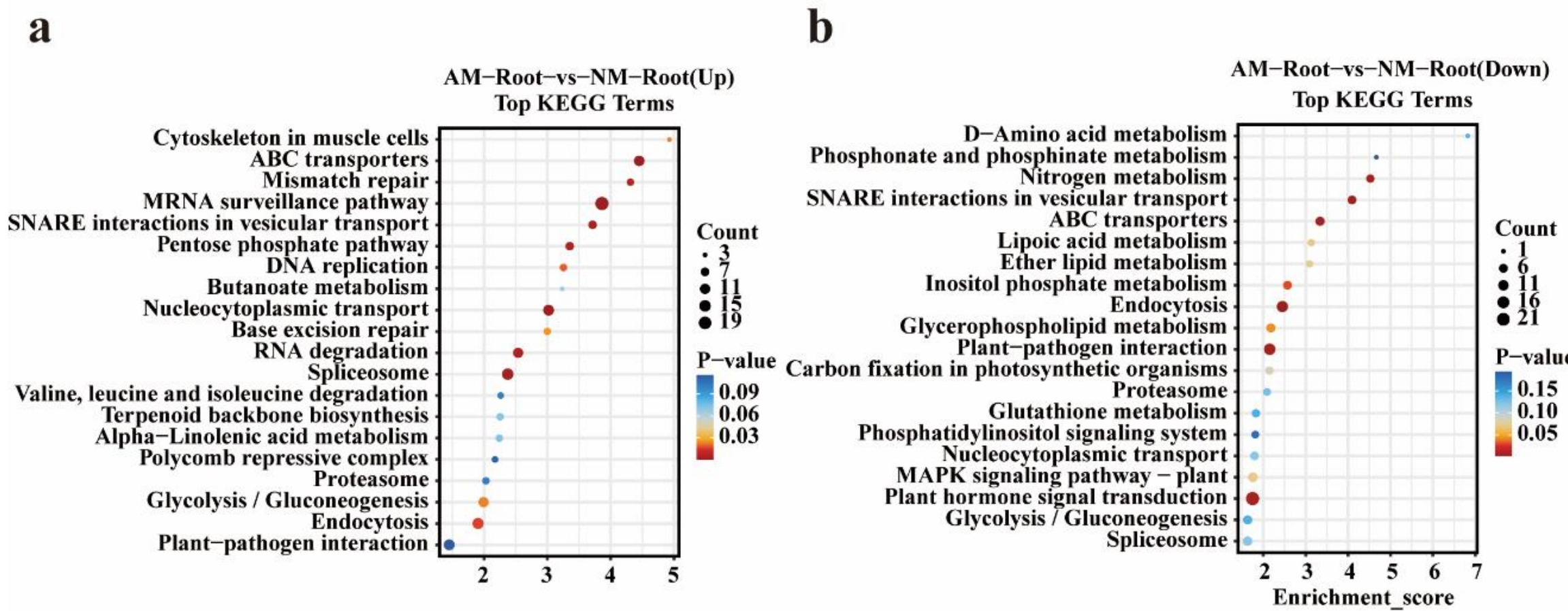

Figure S2. Comparative KEGG pathway analysis of up- (a) and down- (b) regulated phosphoproteins during AM symbiosis. n=4.

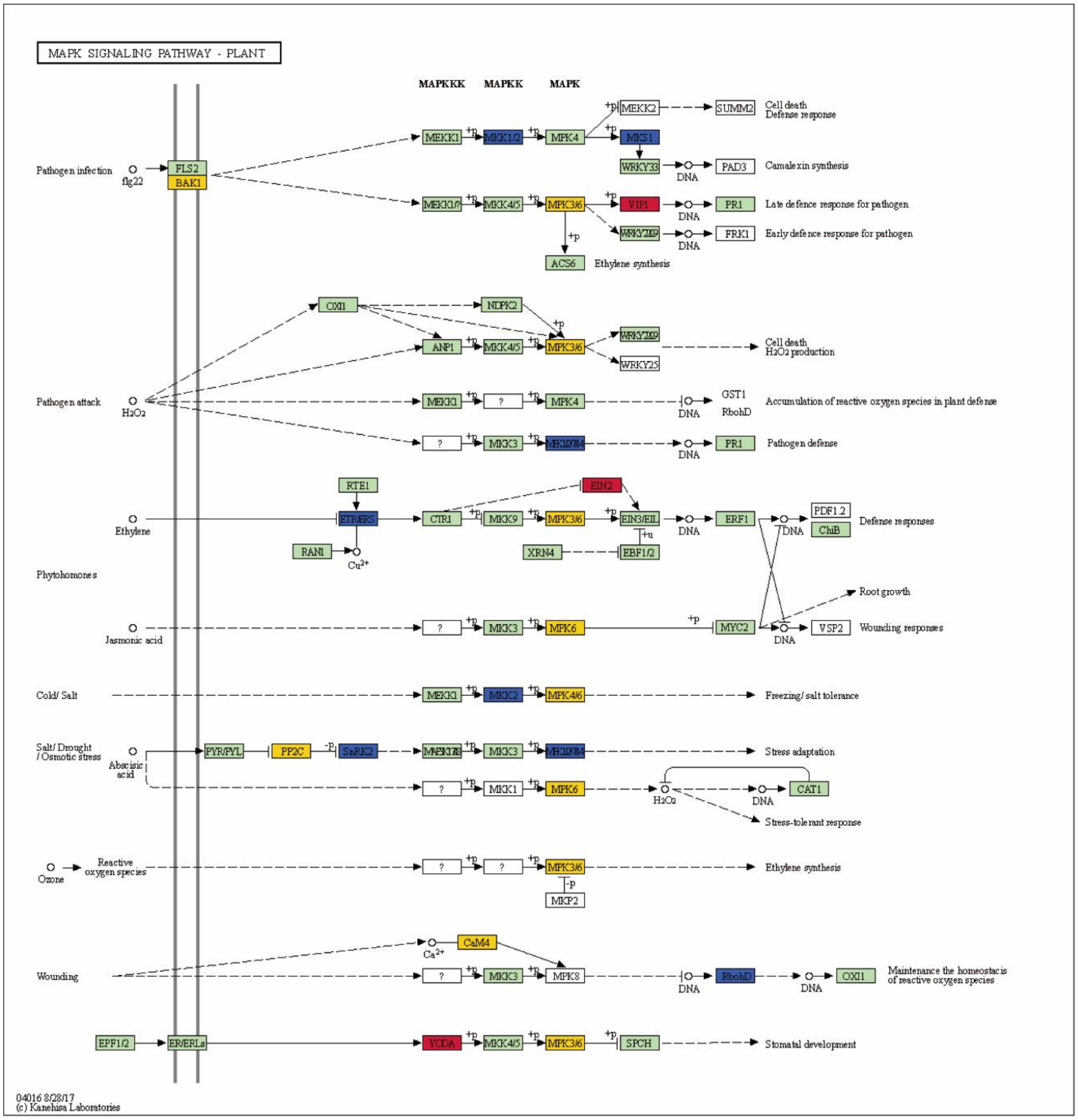

Figure S3. Effects of AM fungal inoculation on the MAPK signaling pathway. Rectangles represent enzymes/genes, with red, blue, and yellow indicating up-regulated, down-regulated, and differentially regulated proteins (both up- and down-regulated), respectively. Uncolored rectangles denote genes present in the MAPK database, whereas light green rectangles highlight species-specific genes.

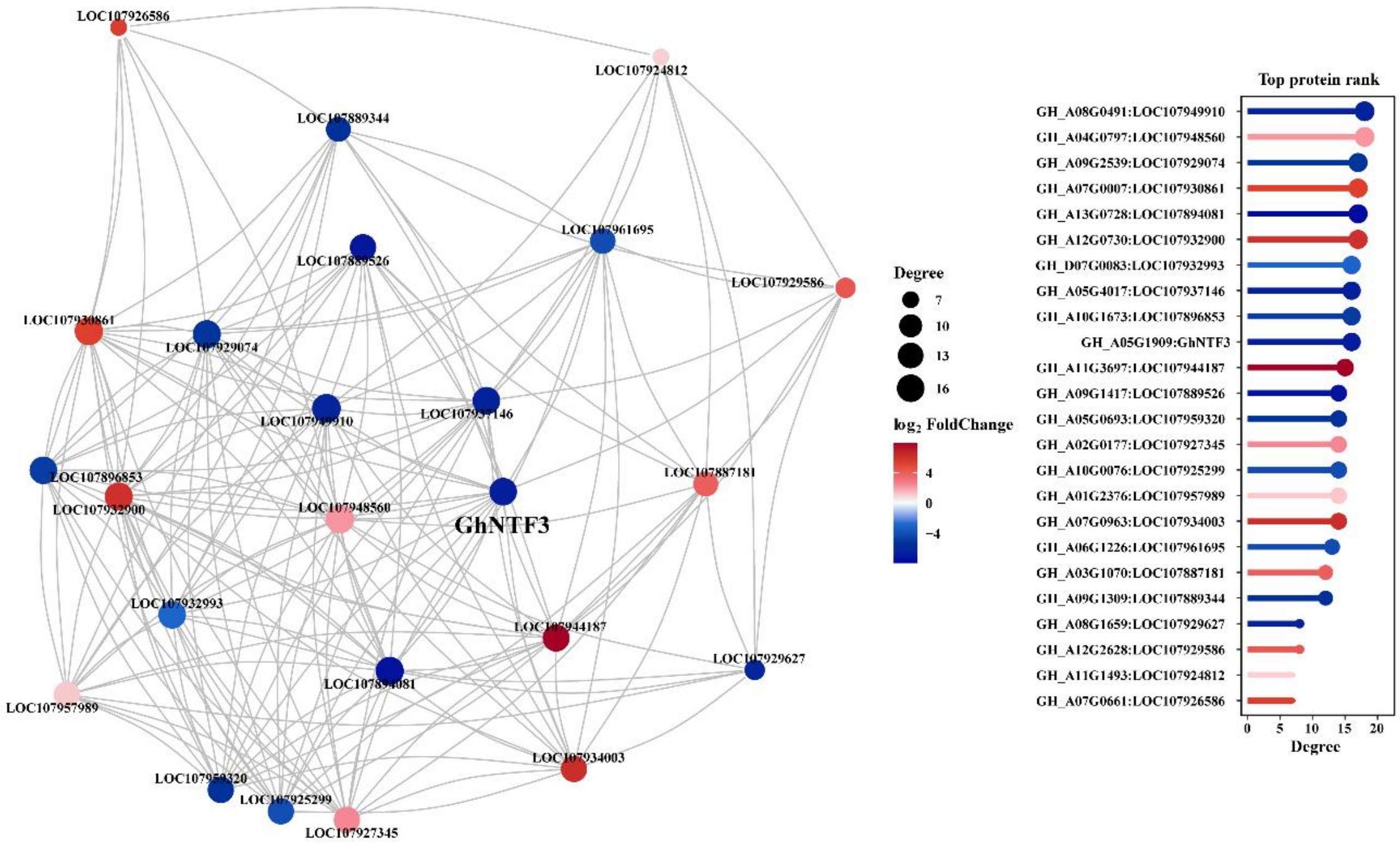

Figure S4. Protein-protein interaction (PPI) network of AM fungus-regulated phosphoproteins. Circular nodes denote differentially phosphoproteins selected based on significance thresholds (|log2FC| > 1, FDR-adjusted $p < 0.05$). Colors indicate red (upregulated) and cyan (downregulated) phosphoproteins. Node size represents topological importance.

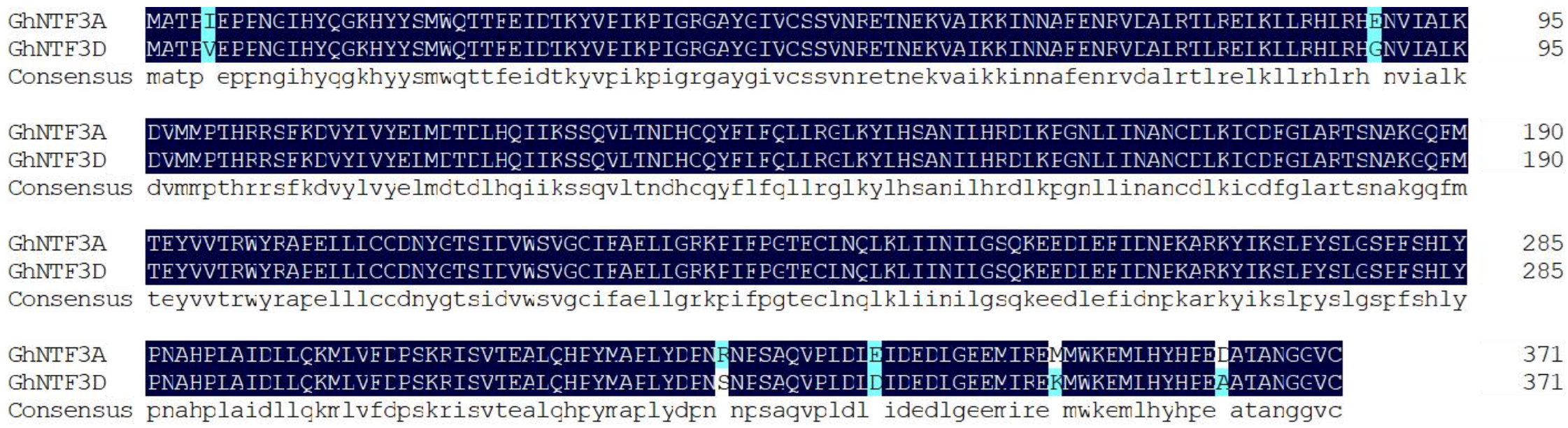

Figure S5. Protein sequence alignment analysis of GhNTF3A and GhNTF3D. GhNTF3A = GH_A051909, GhNTF3D = GH_D05G1947.

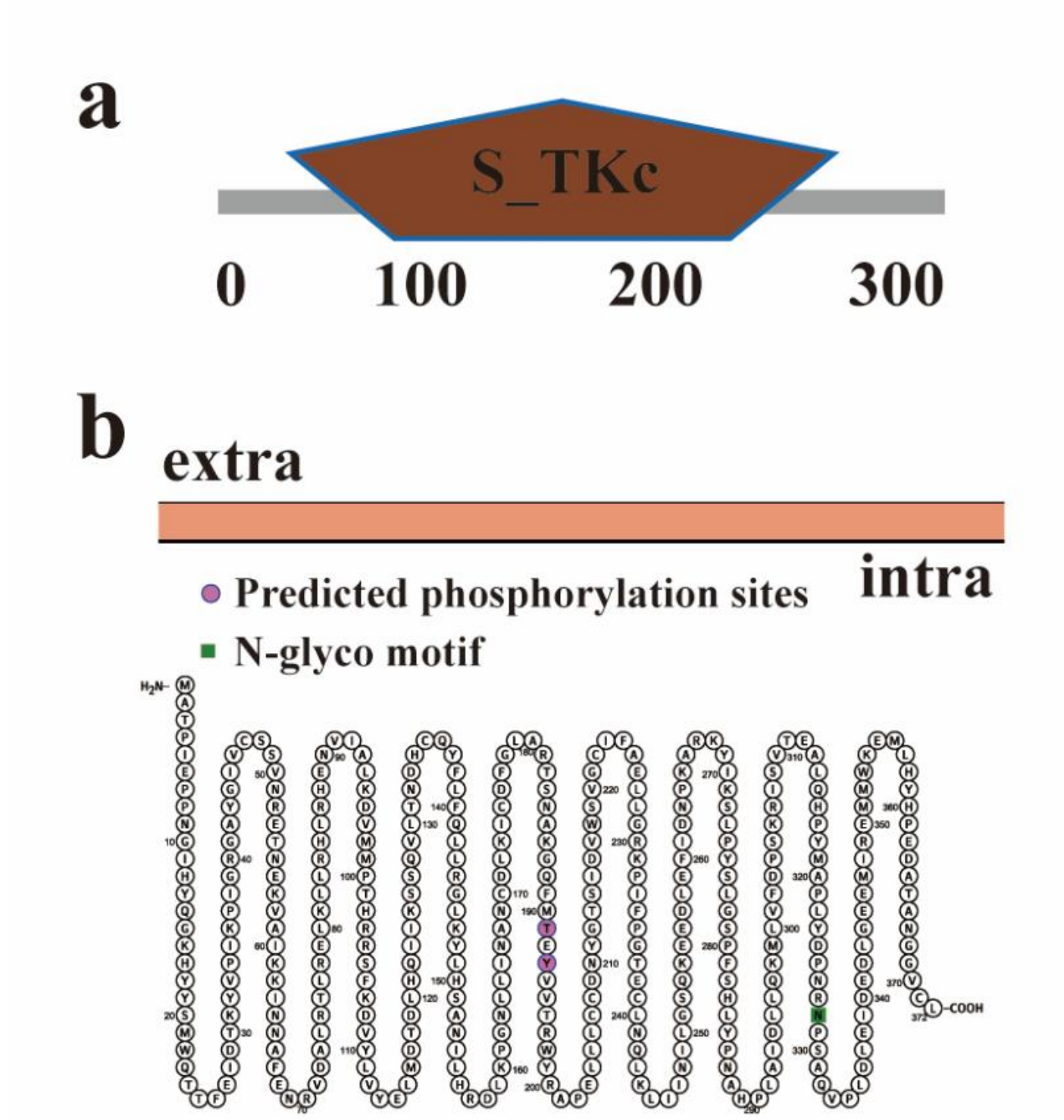

Figure S6. Predicted protein domains (a) and phosphorylation sites (b) of GhNTF3. S_TKc represents the serine/threonine kinase domain. Pink marks indicate the predicted phosphorylation sites.

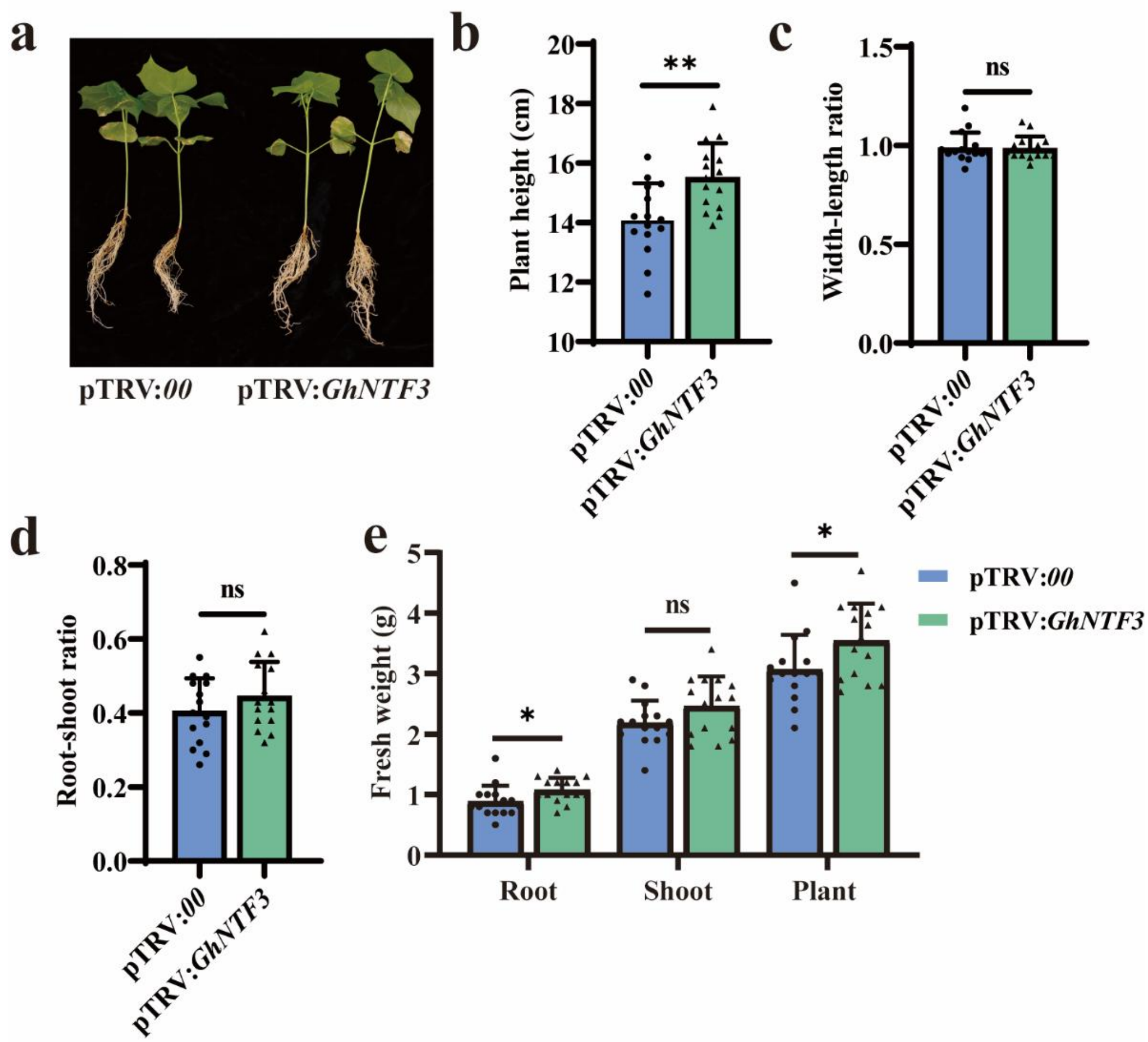

Figure S7. Effect of *GhNTF3* silencing on the growth of mycorrhizal cotton. (a) Phenotypes of *GhNTF3*-silenced cotton seedlings inoculated with AM fungi. At least 15 cotton seedlings were evaluated for plant growth. (b-e) Effects of *GhNTF3* silencing on plant height (b), width-length ratio (c), root-shoot ratio (d), and fresh weight (e) in mycorrhizal cottons. Error bars indicate ±SD of independent replicates. Two-tailed Student' s *t*-test was used to assess statistical differences between pTRV:*00* and pTRV:*GhNTF3* plants. n=15. *, p<0.05; **, p<0.01; ns, not significant.

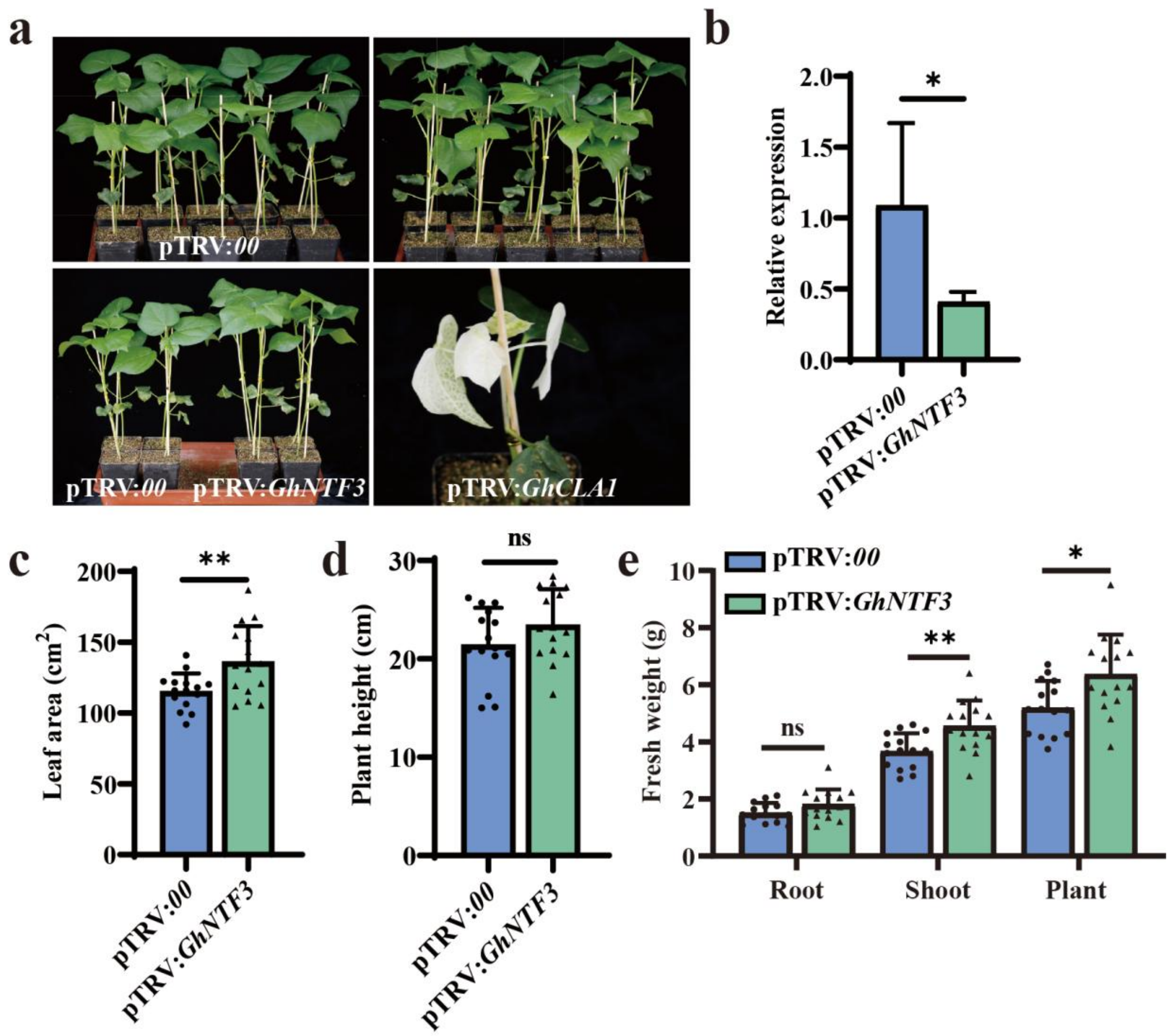

Figure S8. Effects of *GhNTF3* silencing on the growth of non-mycorrhizal cotton. (a) Phenotypes of *GhNTF3*-silenced cotton seedlings without AM fungal inoculation. At least 15 cotton seedlings were evaluated for plant growth. (b-e) Effects of *GhNTF3* silencing on the relative expression of *GhNTF3* (b), leaf area (c), plant height (d), and fresh weight (e) in non-mycorrhizal cottons. Error bars indicate ±SD of independent replicates. Two-tailed *t*-test is used to analyze the difference between pTRV:*00* and pTRV:*GhNTF3* plants. The data points in the bar graphs represent individual biological replicates. Sample sizes were n=5 for gene expression, and n=15 for growth analyses. *, p<0.05; **, p<0.01; ns, not significant.

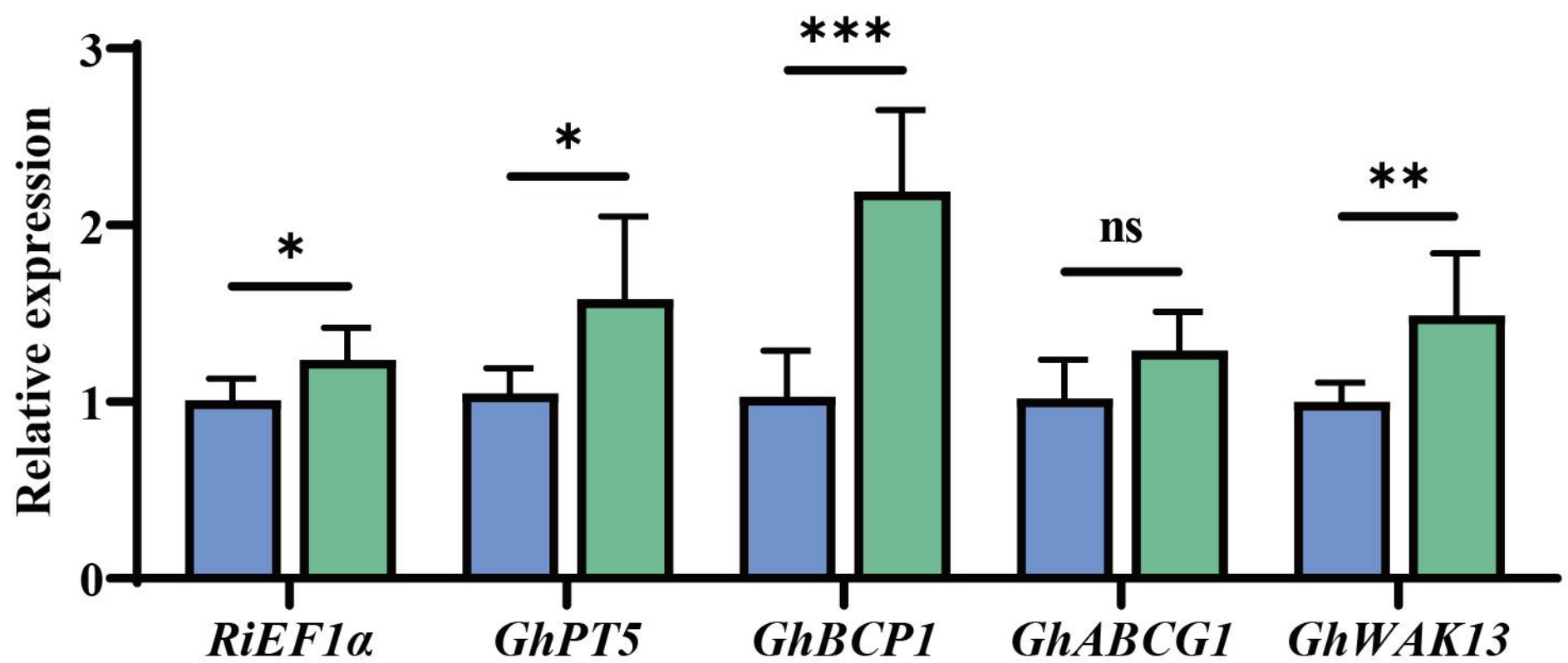

Figure S9. Effects of *GhNTF3* silencing on the relative expression of AM symbiotic marker genes. Blue and green indicate pTRV:*00* and pTRV:*GhNTF3* plants, respectively. Error bars indicate ±SD of independent replicates. Two-tailed *t*-test is used to analyze the difference between pTRV:*00* and pTRV:*GhNTF3* plants. n=6. *, $p<0.05$; **, $p<0.01$; ***, $p<0.001$; ns, not significant.

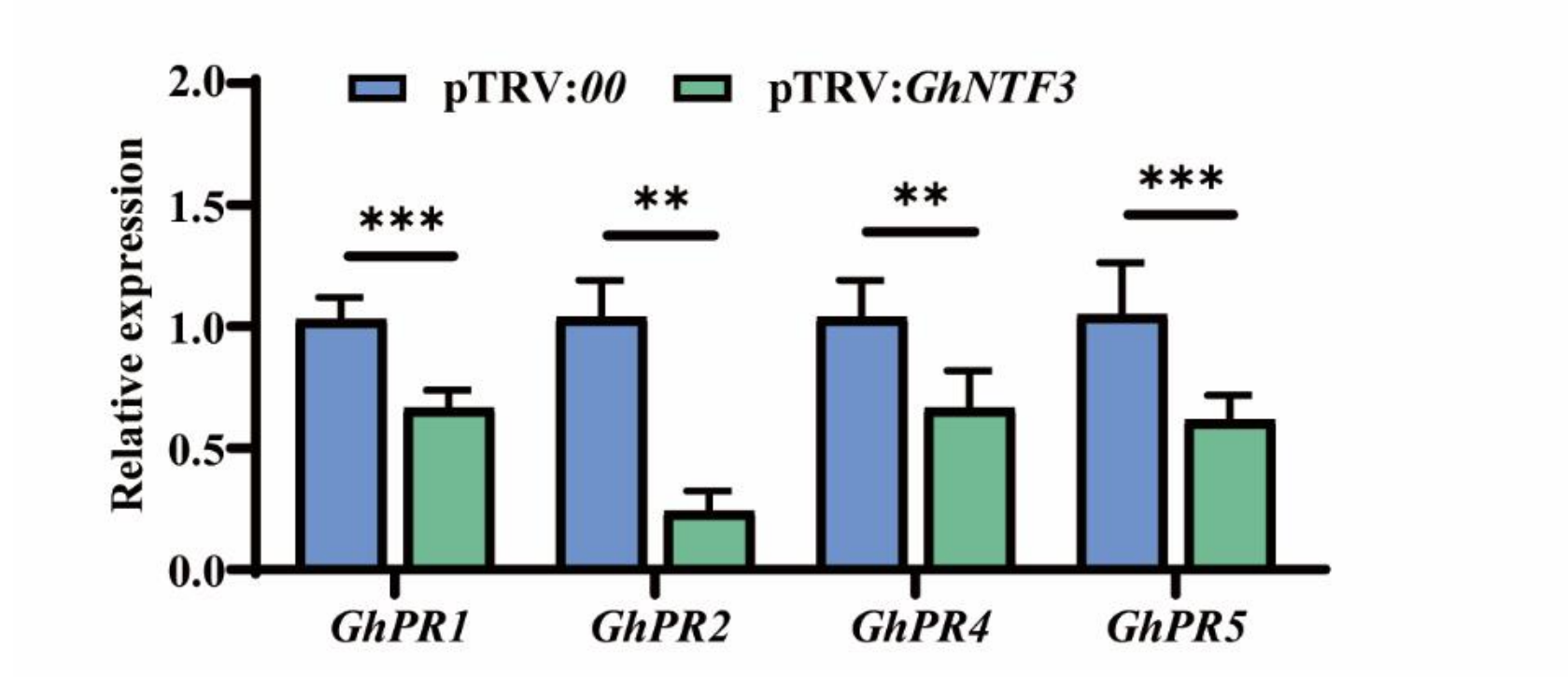

Figure S10. Effect of *GhNTF3* silencing on the expression of pathogenesis-related genes in cotton roots. Error bars indicate ±SD of independent replicates. Two-tailed *t*-test is used to analyze the difference between pTRV:*00* and pTRV:*GhNTF3*. n=6. **, $p<0.01$; ***, $p<0.001$.

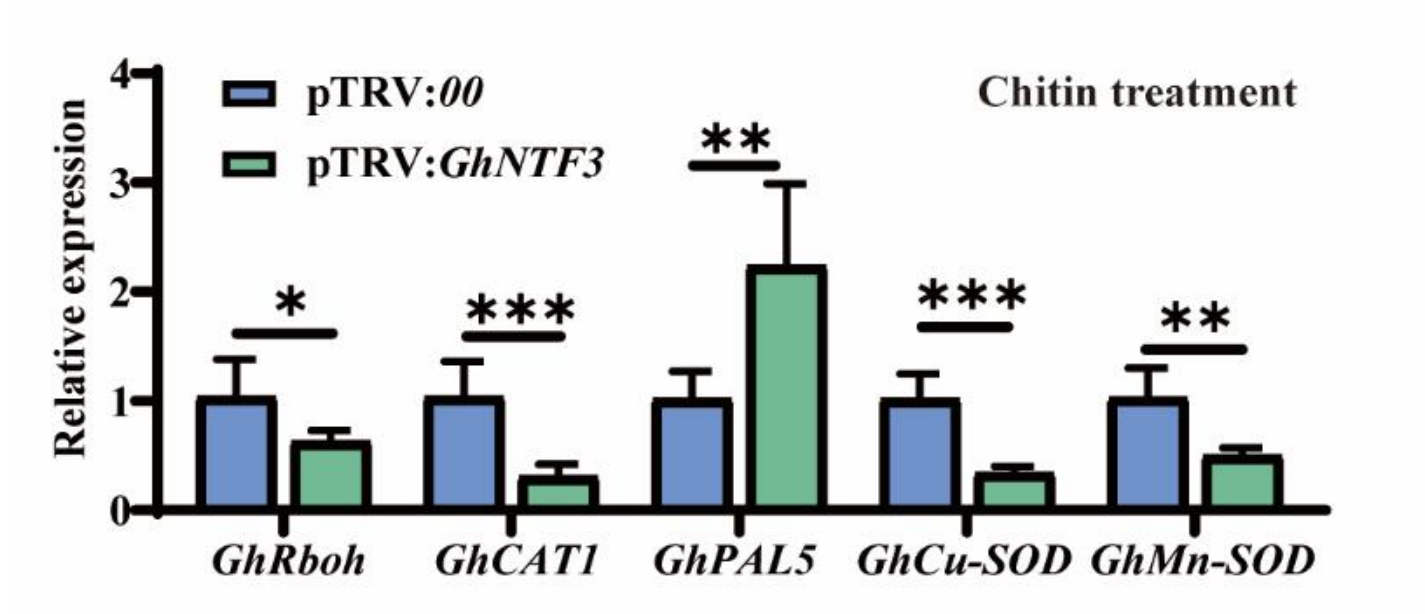

Figure S11. Effect of *GhNTF3* silencing on the expression of disease resistance-related genes under chitin treatment. Error bars indicate ±SD of independent replicates. Two-tailed *t*-test is used to analyze the difference between pTRV:*00* and pTRV:*GhNTF3* plants. n=5. *, p<0.05; **, p<0.01; ***, p<0.001.

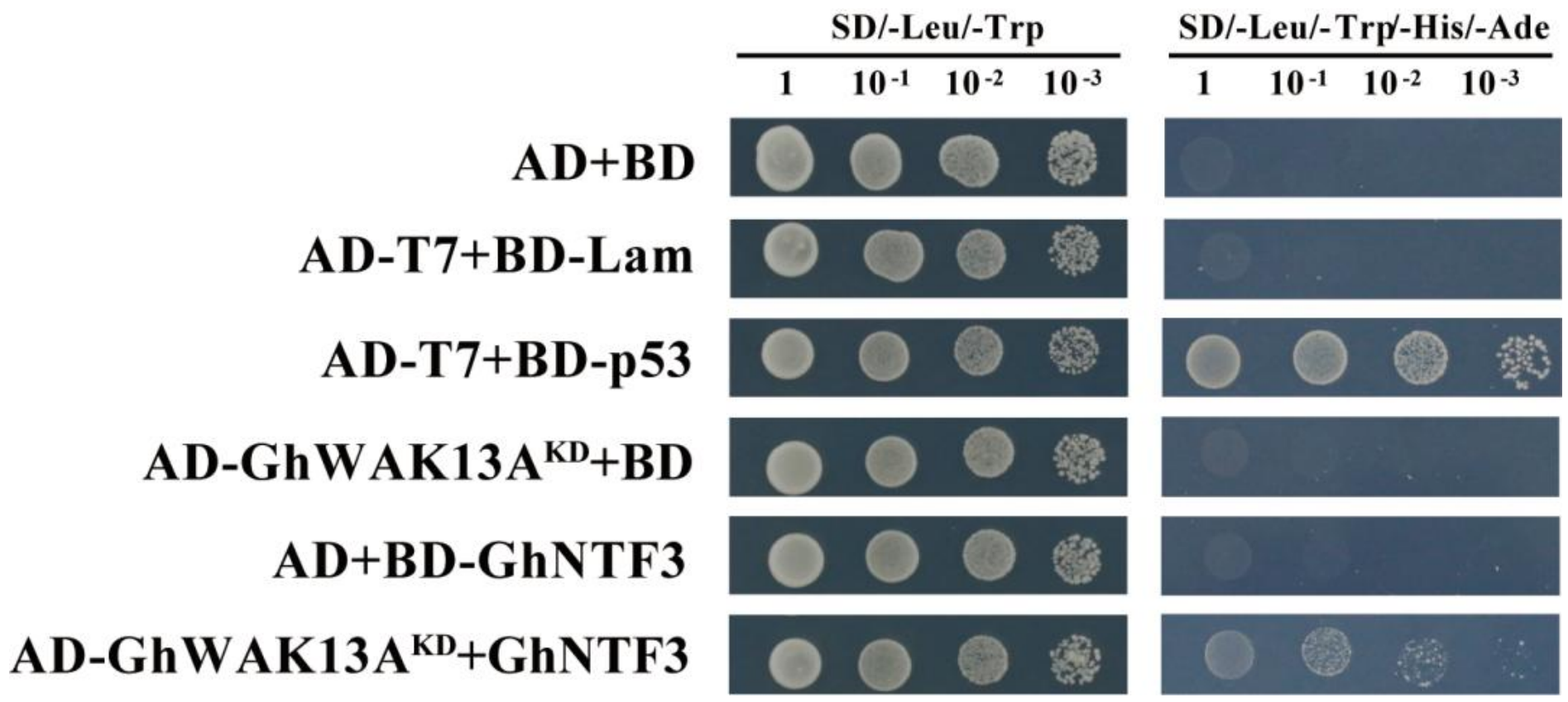

Figure S12. Yeast two-hybrid analysis of the interaction between GhWAK13 and the kinase domain of GhNTF3. KD indicates the kinase domain. 40 mM 3-AT was used to suppress self-activation.

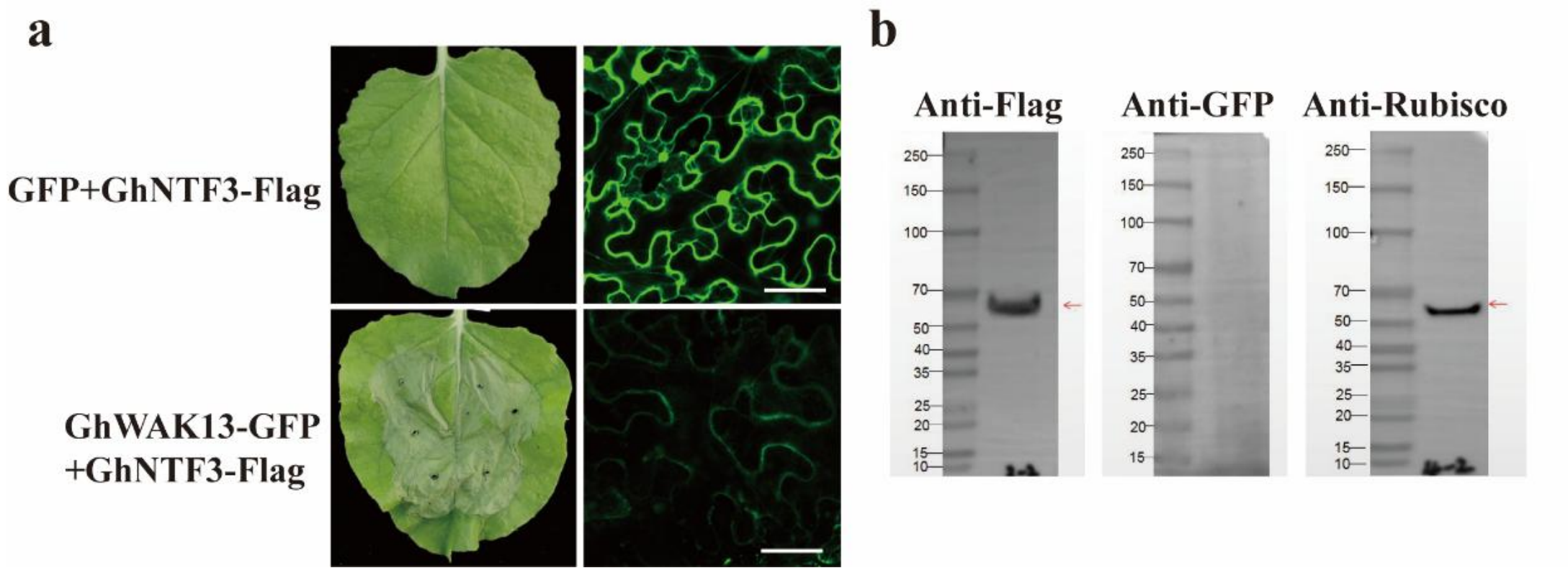

Figure S13. Co-immunoprecipitation analysis of the interaction between GhNTF3 and GhWAK13 in tobacco leaves. (a) Phenotypic observation of tobacco leaves co-expressing GhNTF3 and GhWAK13. Bar=50 μm. (b) Western blot analysis of GhNTF3-Flag and GhWAK13-GFP proteins.

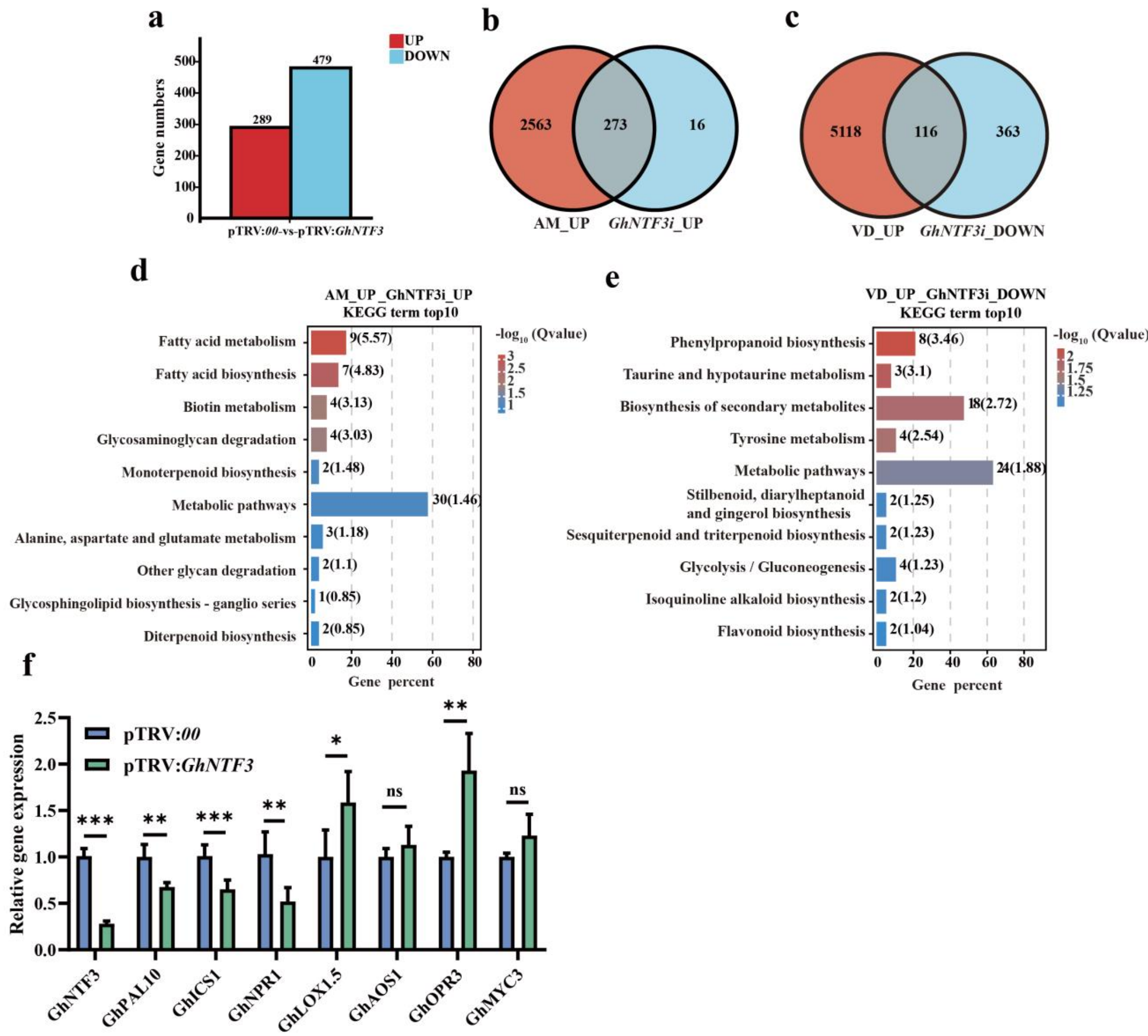

Figure S14. Transcriptomic and hormone analysis of mycorrhizal cottons with silenced *GhNTF3*. (a) Effects of *GhNTF3* silencing on the number of up- and down-regulated genes in cotton roots. Mycorrhizal cotton roots collected 5 weeks inoculation were used for transcriptome analysis. n=5. (b-e) Integrated analysis of the *GhNTF3*-silenced transcriptome with AM symbiosis- or *Verticillium dahliae* responsive transcriptomes. (b) Venn analysis of upregulated genes between *GhNTF3* silencing and AM symbiosis. AM_UP represents AM symbiosis-upregulated genes. GhNTF3_DOWN represents upregulated genes that silenced by *GhNTF3*. (c) Venn analysis of downregulated genes between *GhNTF3* silencing and VD-downregulated genes. VD represents *V. dahliae* infection. GhNTF3_UP represents downregulated genes that silenced by *GhNTF3*. VD_UP represents *V. dahliae* infection upregulated genes. (d) KEGG enrichment analysis of co-upregulated genes by both AM fungi and *GhNTF3* silencing. (e) KEGG enrichment analysis of genes simultaneously upregulated under *V. dahliae* infection and downregulated under *GhNTF3*-silencing. (f) qPCR validation of transcriptome data from *GhNTF3*-silenced plants. *GhUBQ7* was used as a housekeeping gene in cotton. Error bars indicate ±SD of independent replicates. Two-tailed *t*-test is used to analyze the difference between pTRV:00 and pTRV:*GhNTF3* plants. n=5. *, p<0.05; **, p<0.01; ***, p<0.001; ns, not significant.

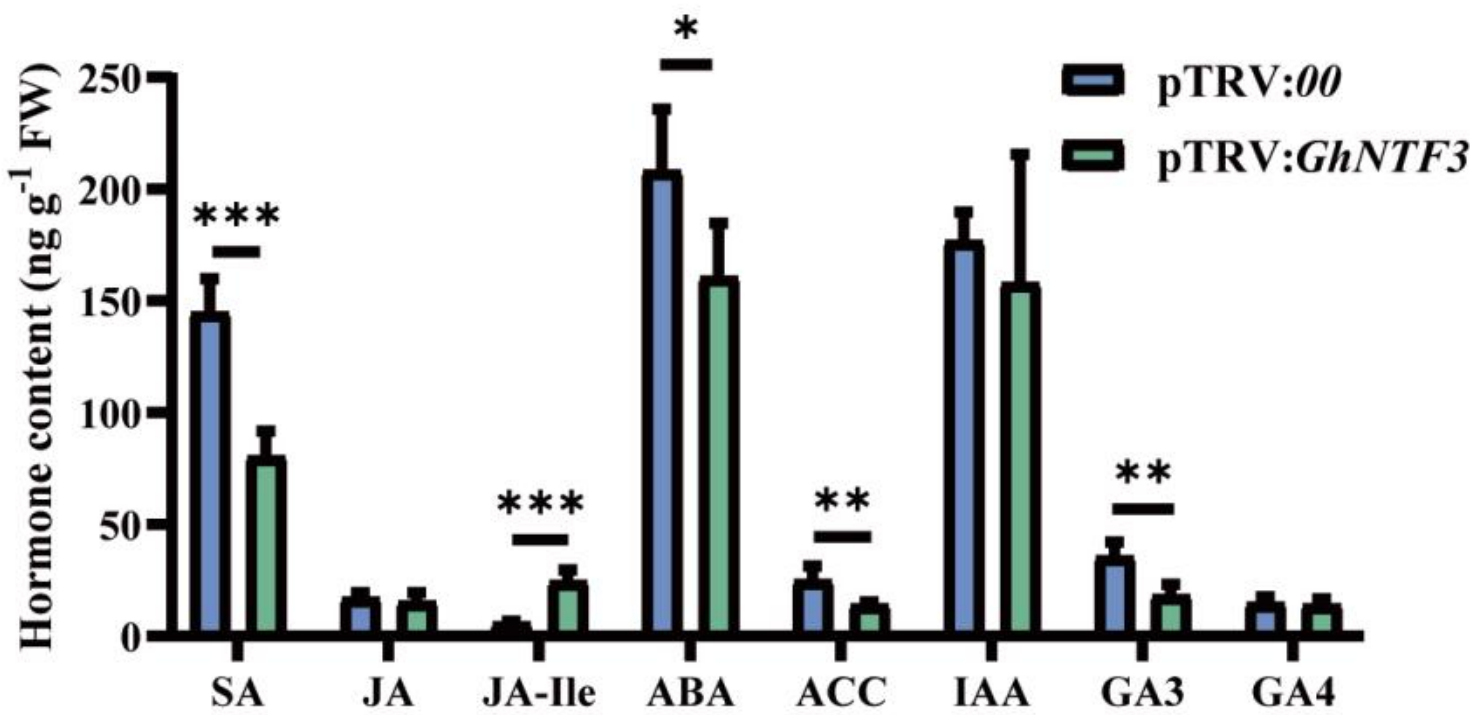

Figure S15. Hormonal changes in non-mycorrhizal roots following *GhNTF3* silencing. SA, salicylic acid; JA, jasmonic acid; JA-Ile, jasmonoyl-isoleucine; ABA, abscisic acid; ACC, 1-aminocyclopropane-1-carboxylic acid; IAA, indole-3-acetic acid; GA, gibberellin. Error bars indicate ±SD of independent replicates. Two-tailed *t*-test is used to analyze the difference between pTRV:00 and pTRV:*GhNTF3*. n=5. *, p<0.05; **, p<0.01; ***, p<0.001.

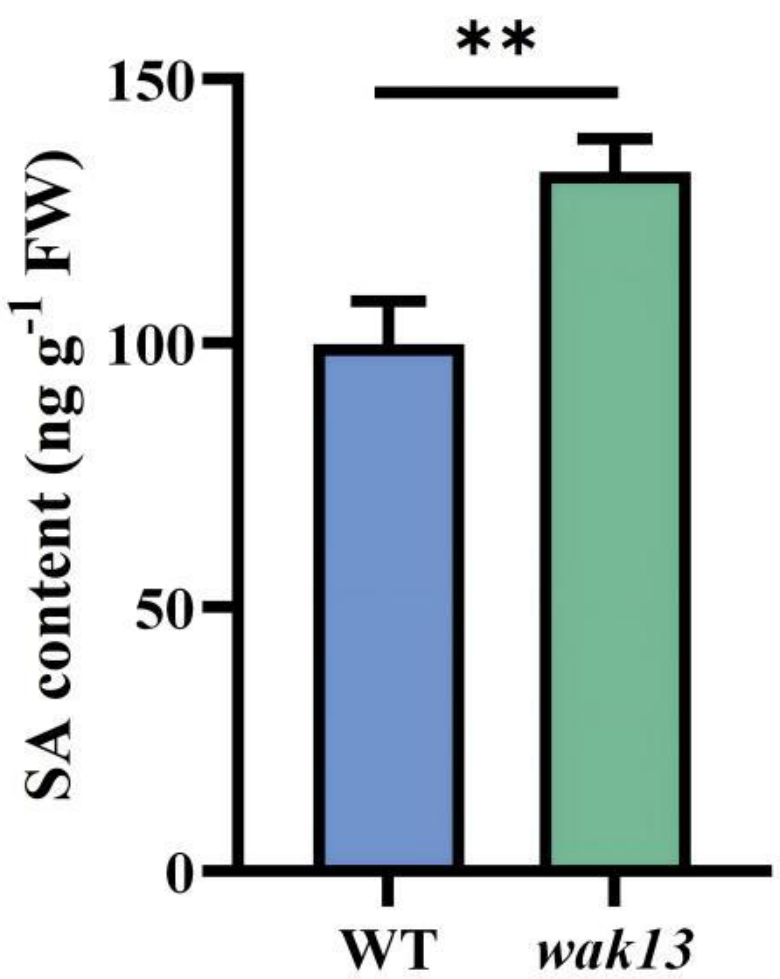

Figure S16. Quantification of SA levels in *GhWAK13*-knockout cotton roots. Error bars indicate ±SD of independent replicates. Two-tailed *t*-test is used to analyze the difference between WT and *wak13* plants. The y-axis represents the salicylic acid content in roots. n=3. **, p<0.01.

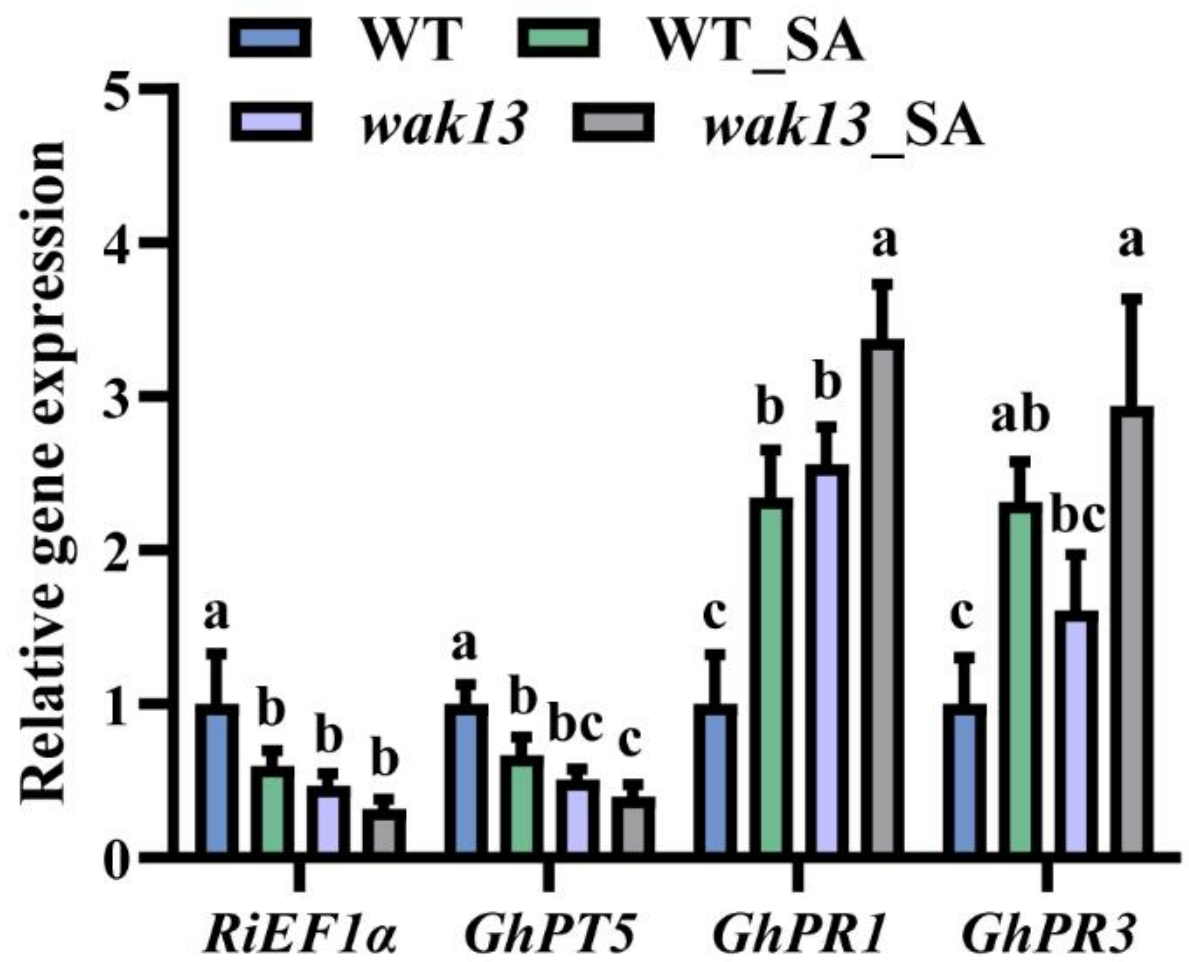

Figure S17. Effects of exogenous SA application on the expression of AM symbiosis-related and pathogenesis-related genes in *GhWAK13*-knockout cotton roots. SA represents exogenous SA application. Error bars indicate ±SD of independent replicates. One-way ANOVA was used to determine statistically significant differences among treatments, followed by Tukey-test post hoc analysis. Each parameter was analyzed separately using ANOVA. Different letters indicate significant differences between each treatment. n=5. p<0.05.

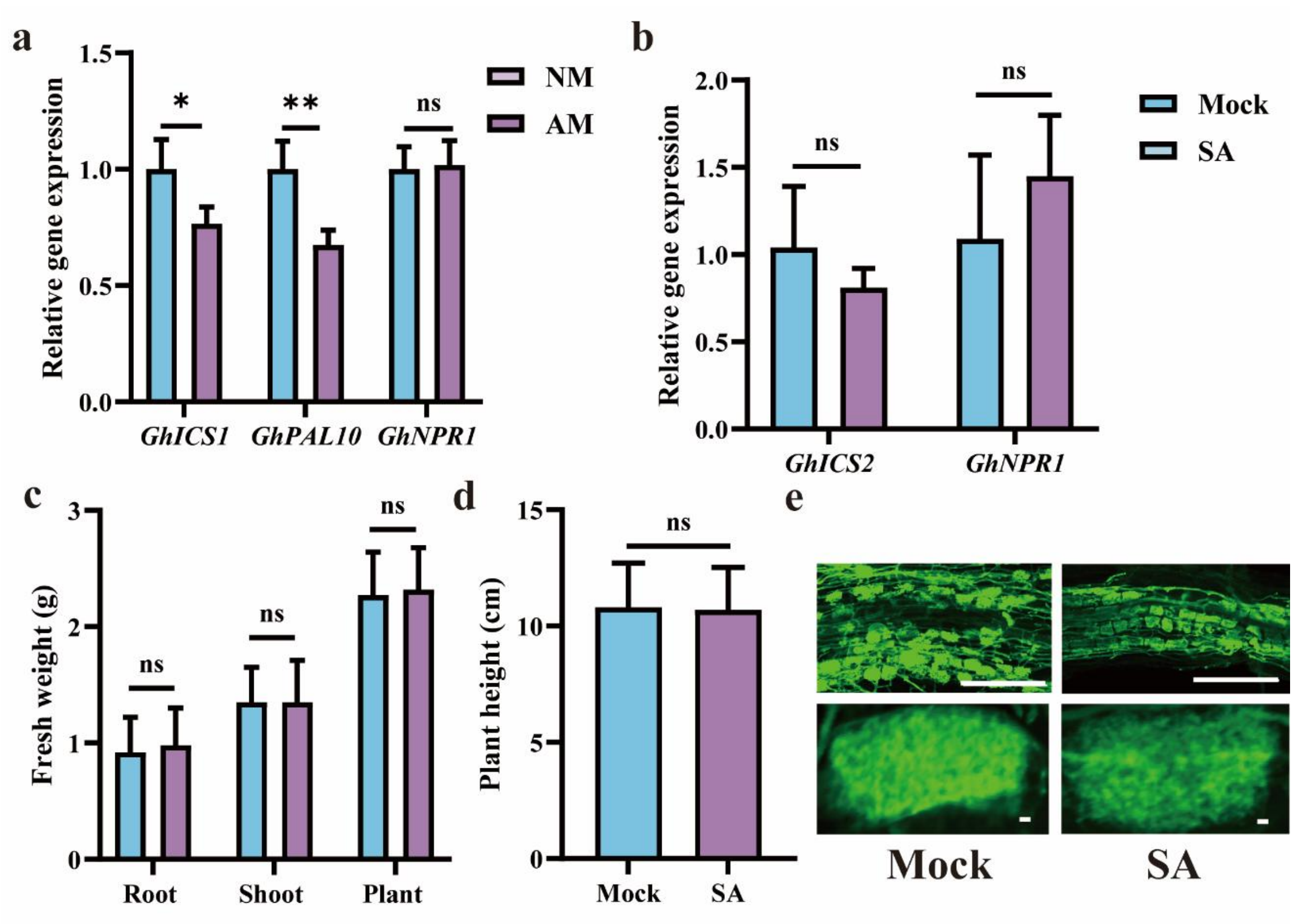

Figure S18. Effects of SA on cotton growth. (a) Expression of SA biosynthesis genes during AM symbiosis. Error bars indicate ±SD of independent replicates. Two-tailed Student’ s *t*-test was used to assess statistical differences between two groups. *GhUBQ7* was used as a housekeeping gene in cotton. $2^{(-\triangle\triangle ct)}$ methods was used to calculate relative expression of these genes. n=4. *, p<0.05; **, p<0.01; ns, not significant. (b-e) Effects of exogenous SA application on the expression of SA biosynthesis genes (b), cotton growth (c), cotton height (d), and colonization structure of AM fungi (e). SA represents exogenous SA application. Error bars indicate ±SD of independent replicates. Two-tailed Student’ s *t*-test was used to assess statistical differences between two groups. For gene expression, n=8. For cotton growth, n=12. ns, not significant. Bar= 200 μm.

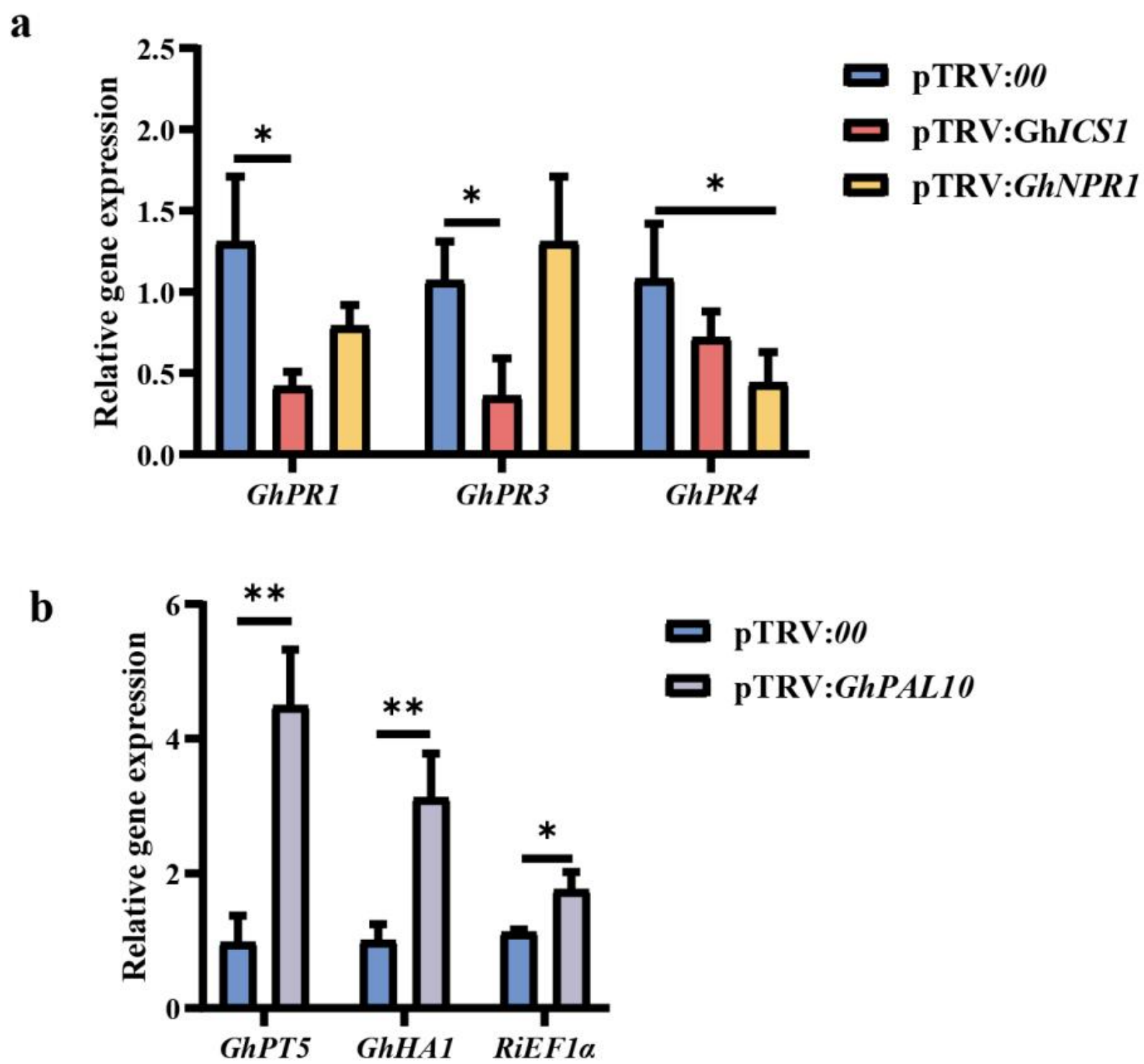

Figure S19. Effects of SA-related genes on gene expression. (a) Effects of *GhICS1* and *GhNPR1* silencing on the expression of pathogen-related genes under AM symbiosis. Error bars indicate ±SD of independent replicates. Two-tailed Student' s *t*-test was used to assess statistical differences between two groups. n=3. *GhUBQ7* was used as a housekeeping gene in cotton. $2^{(-\triangle\triangle ct)}$ methods was used to calculate relative expression of these genes. *, p<0.05; **, p<0.01; ***, p<0.001. (b) Effects of *GhPAL10* silencing on the expression of AM symbiosis-related genes. Error bars indicate ±SD of independent replicate. Two-tailed Student' s *t*-test was used to assess statistical differences between two groups. n=3. *, p<0.05; **, p<0.01.

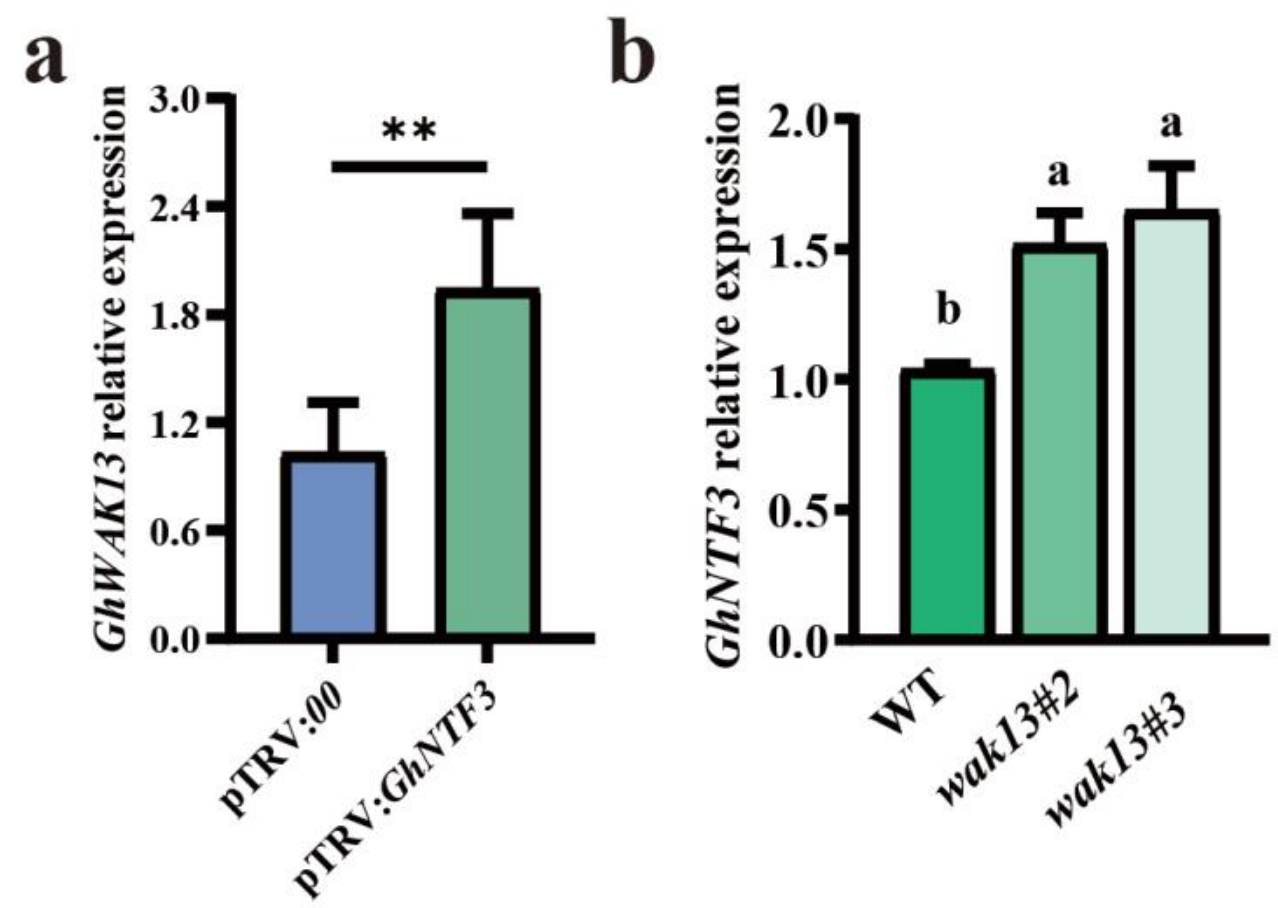

Figure S20. Transcriptional regulation between *GhNTF3* and *GhWAK13*. (a) Expression analysis of *GhWAK13* in *GhNTF3*-silenced cotton roots. Error bars indicate ±SD of independent replicates. Two-tailed Student' s *t*-test was used to assess statistical differences between two groups. n=5. **, p<0.01. (b) Expression analysis of *GhNTF3* in *GhWAK13*-mutanted cotton roots. Error bars indicate ±SD of independent replicates. One-way ANOVA was used to determine statistically significant differences among treatments, followed by Tukey-test post hoc analysis. Different letters indicate significant differences between each treatment. p<0.05. n=3.

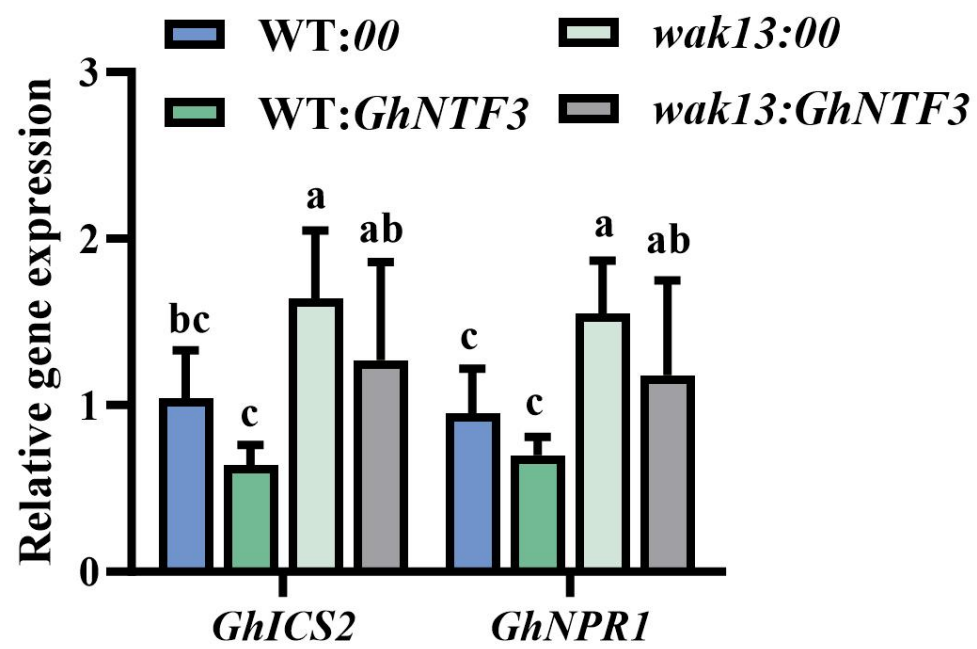

Figure S21. Effects of *GhWAK13* and *GhNTF3* on the expression of SA biosynthetic genes. WT:*00* represents wild-type cotton without *GhNTF3* silencing. *wak13*:*00* represents *GhWAK13*-knockout cotton without *GhNTF3* silencing. *WT*:*GhNTF3* represents wild-type cotton with *GhNTF3* silencing. *wak13*: *GhNTF3* represents *GhWAK13*-knockout cotton with *GhNTF3* silencing. Error bars indicate ±SD of independent replicates. One-way ANOVA was used to determine statistically significant differences among treatments, followed by Duncan-test post hoc analysis. Different letters indicate significant differences between each treatment. ANOVA was performed separately for each gene. n=5. $p<0.05$.

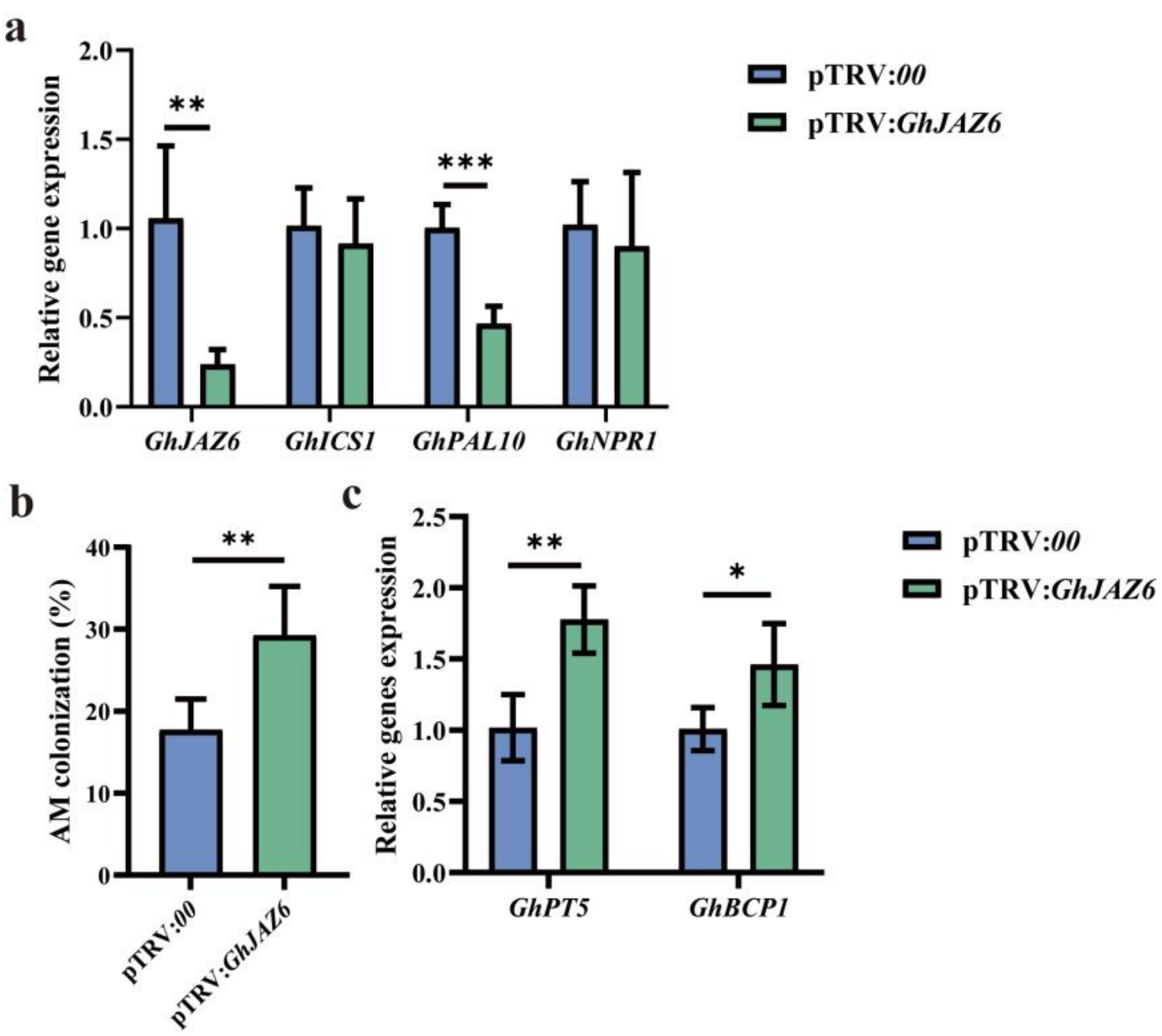

Figure S22. Effects of *GhJAZ6* on SA biosynthetic gene expression and AM fungal symbiosis. (a) Genes involved in SA biosynthesis that regulated by *GhJAZ6*. Error bars indicate ±SD of independent replicates. Two-tailed *t*-test is used to analyze the difference between pTRV:00 and pTRV:*GhJAZ6.* n=4. **, p<0.01; ***, p<0.001. (b-c) Effects of *GhJAZ6* silencing on AM colonization (b) and the transcription of AM symbiotic marker genes (c). Error bars indicate ±SD of independent replicates. Two-tailed *t*-test is used to analyze the difference between pTRV:00 and pTRV:*GhJAZ6.* n=4. *, p<0.05; **, p<0.01.

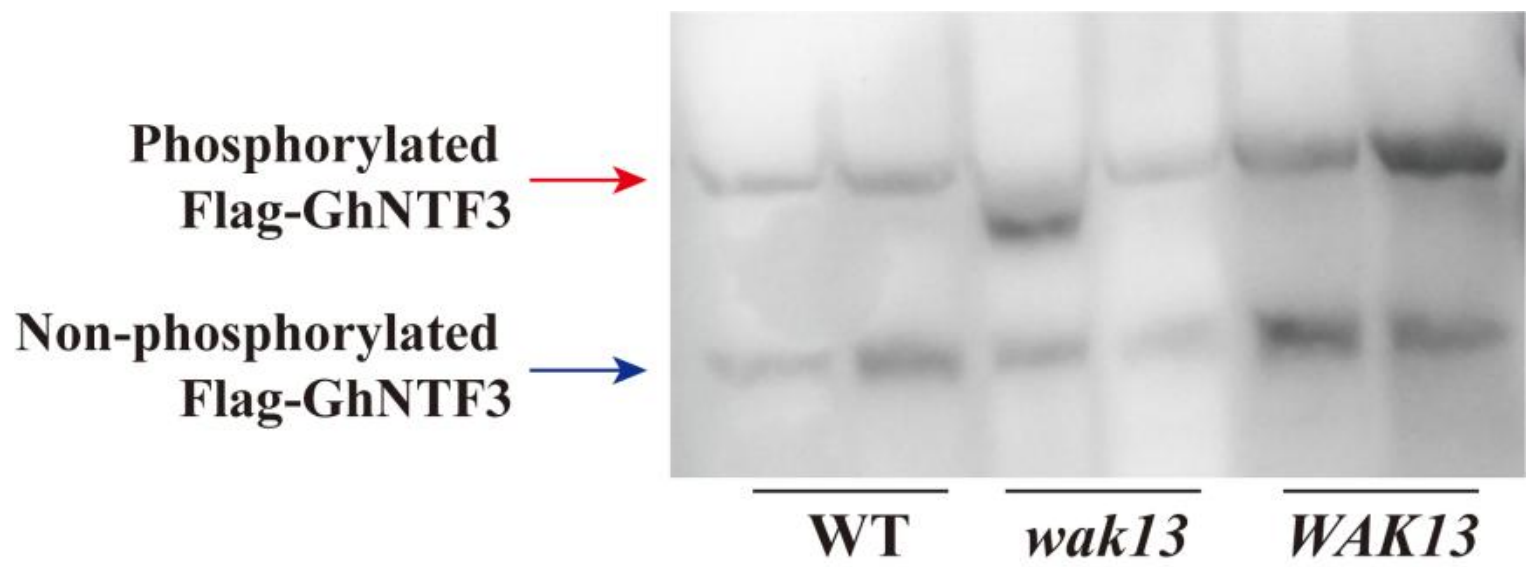

Figure S23. Effects of GhWAK13 on the phosphorylation status of GhNTF3. Red arrows indicate phosphorylated Flag-GhNTF3 bands, while blue arrows indicate non-phosphorylated Flag-GhNTF3 bands. *wak13* denotes *GhWAK13* knockout cotton lines, and *WAK13* denotes *GhWAK13* overexpression lines. This experiment was independently repeated twice.

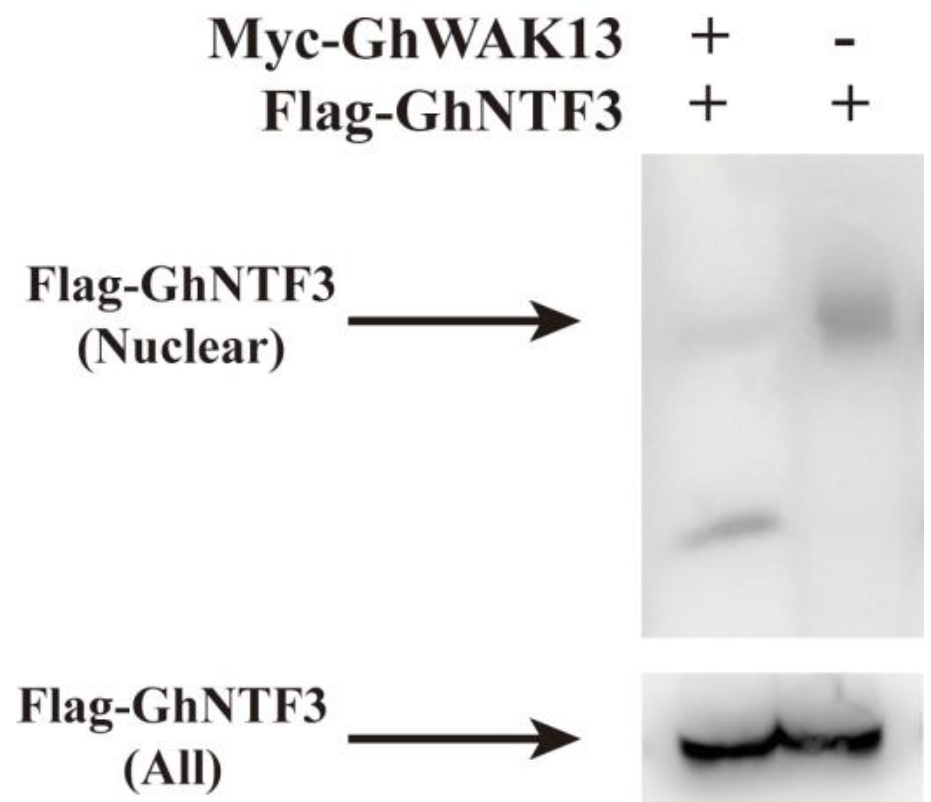

Figure S24. Effects of GhWAK13 on the nuclear/cytoplasmic distribution of GhNTF3. Constructs carrying Myc-GhWAK13 and Flag-GhNTF3 were co-infiltrated into tobacco leaves. Total proteins and nuclear protein fractions were separately extracted, and Flag-GhNTF3 levels were detected using an anti-Flag antibody. This experiment was independently repeated twice.

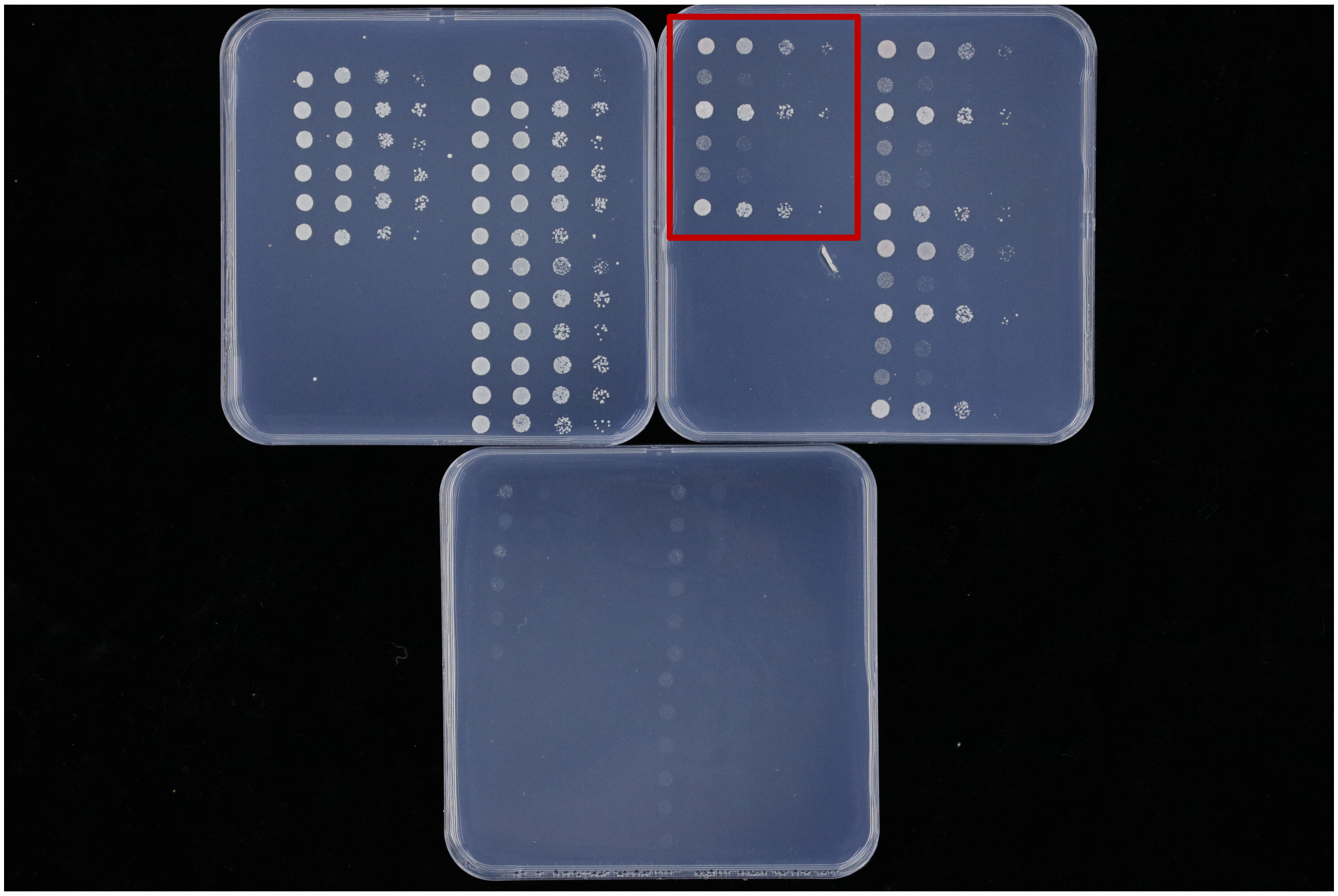

Figure S25. The original image showing the yeast two-hybrid interaction between GhWAK13 and GhNTF3. The red square marks the region excerpted from the article.

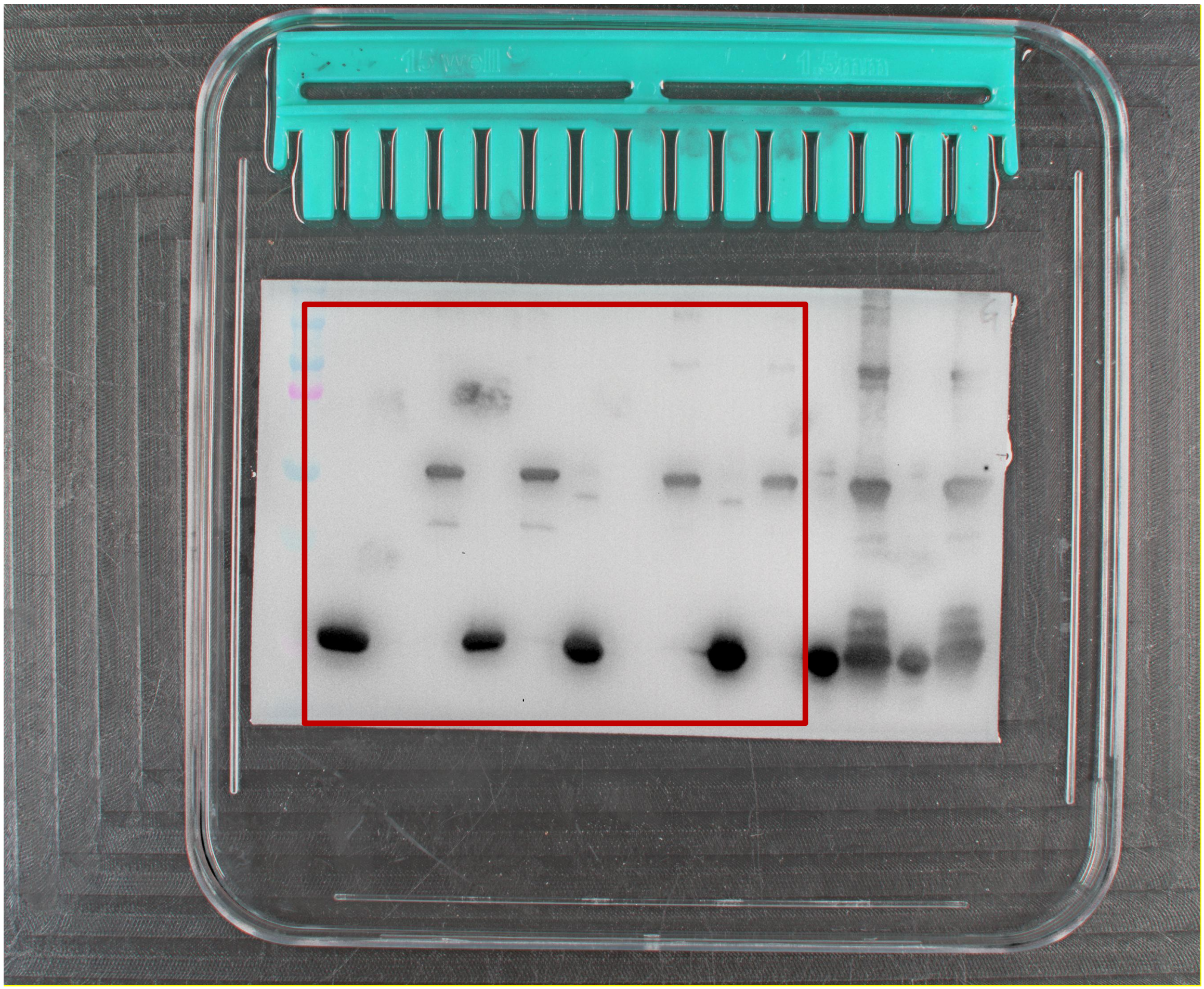

Figure S26. Original pull‑ down image for in vitro interaction between GhWAK13 and GhNTF3. The red square marks the region excerpted from the article..

Table S1. Yeast two-hybrid screening results of GhNTF3

Gene ID	Gene name	Gene description
GH_A05G2392	GhWAK13	Wall-associated kinase 2
GH_A06G0856	GhJAZ6	Jasmonate-zim-domain protein 1
GH_A12G2591	GhMAP4K6	Protein kinase superfamily protein
GH_D08G1341	WRKY41	WRKY family transcription factor
GH_D11G1438	CYP75B1	Cytochrome P450 superfamily protein
GH_A08G2285	GhPP2C25	Protein phosphatase 2C family protein
GH_A02G1059	GhNLP7	NIN like protein 7

Dataset S1 (separate file). AM fungus-regulated phosphorylated peptides.
Dataset S2 (separate file). All primers used in this study.
Dataset S3 (separate file). All raw data in this study.
